# Chronic myelogenous leukemia cells remodel the bone marrow niche via exosome-mediated transfer of miR-320

**DOI:** 10.7150/thno.34813

**Published:** 2019-07-28

**Authors:** Xiaotong Gao, Zhuo Wan, Mengying Wei, Yan Dong, Yingxin Zhao, Xutao Chen, Zhelong Li, Weiwei Qin, Guodong Yang, Li Liu

**Affiliations:** 1 Department of Hematology, Tangdu Hospital, Fourth Military Medical University, Xi'an, 710038, People's Republic of China.; 2 State Key Laboratory of Cancer Biology, Fourth Military Medical University, Xi'an, 710032, People's Republic of China.; 3 Department of Biochemistry and Molecular Biology, Fourth Military Medical University, Xi'an, 710032, People's Republic of China.; 4 Department of Implantation, School of Stomatology, Fourth Military Medical University, Xi'an, 710032, People's Republic of China.; 5 Department of Ultrasound Diagnostics, Tangdu Hospital, Fourth Military Medical University, Xi'an, 710038, People's Republic of China.

**Keywords:** chronic myelogenous leukemia, exosomes, bone marrow mesenchymal stromal cells, miR-320, HNRNPA1

## Abstract

**Rationale:** Reciprocal interactions between leukemic cells and bone marrow mesenchymal stromal cells (BMMSC) remodel the normal niche into a malignant niche, leading to leukemia progression. Exosomes have emerged as an essential mediator of cell-cell communication. Whether leukemic exosomes involved in bone marrow niche remodeling remains unknown.

**Methods:** We investigated the role of leukemic exosomes in molecular and functional changes of BMMSC *in vitro* and *in vivo*. RNA sequencing and bioinformatics were employed to screen for miRNAs that are selectively sorted into leukemic exosomes and the corresponding RNA binding proteins.

**Results:** We demonstrated that leukemia cells significantly inhibited osteogenesis by BMMSC both *in vivo* and *in vitro*. Some tumor suppressive miRNAs, especially miR-320, were enriched in exosomes and thus secreted by leukemic cells, resulting in increased proliferation of the donor cells. In turn, the secreted exosomes were significantly endocytosed by adjacent BMMSC and thus inhibited osteogenesis at least partially via β-catenin inhibition. Mechanistically, miR-320 and some other miRNAs were sorted out into the exosomes by RNA binding protein heterogeneous nuclear ribonucleoprotein A1 (HNRNPA1), as these miRNAs harbor the recognition site for HNRNPA1.

**Conclusion:** HNRNPA1-mediated exosomal transfer of miR-320 from leukemia cells to BMMSC is an important mediator of leukemia progression and is a potential therapeutic target for CML.

## Introduction

Chronic myelogenous leukemia (CML) results from the transformation of normal hematopoietic stem cells (HSC) by the BCR-ABL oncogene. Disruption of normal hematopoiesis occurs even before outgrowth of leukemic cells, causing anemia, thrombocytopenia, neutropenia and osteoporosis, which are the major causes of morbidity and mortality of CML. The bone marrow hematopoietic microenvironment (“niche”) is a unique composition of mesenchymal and non-mesenchymal cells, which supports the self-renewal of normal and malignant hematopoietic cells 1. Reciprocal interactions between leukemic cells and the niche are essential for the leukemic progression and resistance to drug treatment 1. In addition, emerging evidence has shown that myeloid malignancies induce molecular and functional changes in the niche 2-5, switching the normal hematopoietic cell favouring niche to leukemic cell favouring one 6-8. However, the mechanisms by which leukemic cells remodel the bone marrow niche have not been adequately defined.

Exosomes are recognized as pivotal mediators of cell-cell communication, through transmission of intracellular cargo such as miRNAs 9, 10. Exosomes loaded with miRNAs are secreted by cancer cells and internalized by neighbouring cells in the malignant niche, affecting the phenotype of niche cells 11-15. Specific miRNAs are selectively sorted into exosomes, through mechanisms that are still poorly understood 16. It has been reported that RNA-binding proteins such as heterogeneous nuclear ribonucleoproteins A2B1 (HNRNPA2B1) and Q (HNRNPQ) are involved in exosomal RNA export by recognizing and binding specific motifs in miRNA sequences 17, 18. Notably, HNRNPA1 is induced by BCR/ABL tyrosine kinase in a dose- and kinase-dependent manner 19. In addition, HNRNPA1 controls the nuclear export of mRNAs that are indispensable for the leukemic phenotype of CML 20. All of these suggest that leukemic cells might selectively sort certain miRNAs into the exosomes during the progression of CML.

Here, by using leukemic cell lines, a murine leukemic model, and patient samples, we found that leukemic cells selectively transport growth-suppressing miRNAs into exosomes via heterogeneous nuclear ribonucleoprotein A1 (HNRNPA1), resulted in promoted CML cell growth. In addition, the CML cell-derived exosomes could be endocytosed by the surrounding BMMSC, leading to inhibited osteogenesis of the recipient cells and thus remodelled bone marrow niche favourable for CML progression.

## Materials and methods

### Animals

Double-transgenic SCL-tTa X TER-BCR/ABL mice 21 were backcrossed for at least eight generations into the C57BL/6 background and were maintained in the presence of 0.5 g/L doxycycline in drinking water. BCR/ABL expression was induced in 5-week-old double-transgenic mice (BA) by doxycycline withdrawal for at least 4 weeks. All mice were maintained under specific pathogen free conditions and the experimental procedures were performed in accordance with guidelines and protocols approved by the Institutional Animal Experiment Administration Committee of Fourth Military Medical University.

### Cell lines and human samples

Human CML cell lines K562 and LAMA84 were purchased from ATCC and cultured in RPMI 1640 medium (Hyclone, Loga, UT, USA) with 10% FBS and 1% penicillin-streptomycin (Hyclone).

Human bone marrow and peripheral blood specimens were obtained from 41 patients with newly diagnosed CML; 24 patients were in chronic phase (CML-CP) and 17 patients in blast crisis (CML-BC). Specimens from 11 age-matched healthy donors were used as control. All subjects signed an informed consent form. All specimen acquisition was approved by the Ethics Committee of Fourth Military Medical University.

### Isolation and expansion of bone marrow mesenchymal stromal cells (BMMSC)

BMMSC were isolated from the bone marrow of CML-CP patients and age-matched healthy donors after informed written consent as previously described 22. Briefly, the mononuclear cell fraction was isolated by density gradient centrifugation using Human Lymphocyte Separation Medium (Dakewe Biotech, Shenzhen, China), re-suspended in alpha-MEM (Gibco, Carlsbad, CA, USA) containing 5% human platelet lysate (HELIOS, Atlanta, GA, USA), 2 mM L-glutamine, and 1% penicillin-streptomycin (Hyclone), and plated at an initial density of 1×10^6^ cells/cm^2^. Three days later, non-adherent cells were removed and the remaining monolayers of adherent cells were cultured in fresh medium until they reached confluence. The cells were harvested by trypsinization and sub-cultured at densities of 5000-6000 cells/cm^2^. BMMSC were identified as CD34-/CD45-/HLA-DR-/CD29+/CD90+/CD105+ using flow cytometry. Multipotent differentiation capacity was demonstrated by osteogenic, adipogenic and chondrogenic induction as described below. For osteogenesis analysis, BMMSC cells at third to fifth passages were used. For miR-320a expression analysis in the BMMSCs, the primary culture was used.

### Exosome purification and characterization

For exosome isolation from K562 and LAMA84 cells, RPMI 1640 medium with 10% exosome-depleted FBS (ultracentrifugation at 120,000g for 16 h) was used. For exosome isolation from mice or patients' hematopoietic cells, bone marrow mononuclear cells were collected from either mice femurs or patients' bone marrow samples and then cultured using StemSpan SFEM II medium (Stemcell Technologies, Vancouver, Canada) without FBS. Exosomes were isolated by differential centrifugation. In detail, supernatant fractions collected from 48-72 hr cell cultures were centrifuged at 800g for 5 min and 2000g for 10 min. The supernatant was then filtered using 0.22 μm filters, followed by a final centrifugation at 10,0000g for 2 hr. The supernatant was aspirated and the pellet was re-suspended in PBS. The concentration of exosomes was determined by total exosomal proteins using the Pierce BCA Protein Assay Kit (Thermo Scientific, Waltham, MA, USA), according to the manufacturer's instruction. For exosome isolation from serum, serum was collected from all blood samples using a 2-step centrifugation protocol (2000g for 10 min, 10000g for 10 min) to remove cellular debris. Serum was then incubated with ExoQuick^TC^ exosome precipitation solution (System Biosciences, Johnstown, PA, USA) according to manufacturer's instructions. Exosomes were stored at 4°C and analyzed or used for experimental procedures within one week after harvest.

NanoSight and transmission electron microscopy were used to determine exosome size distribution, concentration and morphology. For biomarker analysis of exosomes, the exosome pellet was dissolved in lysis buffer for western blot (see below).

### Colony forming unit-fibroblast assay

The colony forming unit-fibroblast (CFU-F) assay was performed as previous described 23. Briefly, normal or BA mice were sacrificed by CO_2_ and their femurs were cleaned from muscle and crushed into pieces to release bone marrow cells. The marrow-free bone pieces were further digested with 3% collagenase (Type I, Sigma-Aldrich, Saint Louis, MO USA) at 37 ^o^C for 40 min. 2 x 10^4^ cells were plated into a 60 mm dish in MesenCult^TM^ Expansion Medium (Stemcell Technologies). The medium was removed after 14 days culture and cells were fixed with 4% PFA for 5 min at room temperature and then stained with crystal violet for 15 min. After washing, colonies with more than 50 cells per colony were counted.

### Quantitative real-time PCR

Total RNA from cells or exosomes was extracted with Tripure Isolation Reagent (Roche, Basel Switzerland) following the manufacturer's instructions. RNA purity and concentration were assessed with a Nanodrop-1000 spectrophotometer (Thermo Fisher Scientific, Waltham, MA, USA). miRNA expression was measured by quantitative real-time PCR (qPCR) using miRcute miRNA First-Strand cDNA Synthesis Kit and miRcute miRNA SYBR Green qPCR Detection Kit (Tiangen, Beijing, China). U6 was used as an internal control. For analysis of mRNA expression, 1 μg total RNA was reverse transcribed using Transcriptor Reverse Transcriptase Kit (Roche) and qPCR was performed using FastStart Essential DNA Green Master Kit (Roche). GAPDH was used for normalization. All kits were used per manufacturers' instructions. The primer sequences are listed in Table S1. qPCR was run using the Roche LightCycler 96 qPCR system (Roche) with each reaction run in triplicate. The change in miRNA or mRNA expression was calculated by normalizing to the control sample as described previously 24.

### Co-culture assay

K562 cells pre-transfected with 50 nM C. elegans specific miRNA (*cel*-miR-39) were placed on the insert of a trans-well plate (0.4 μm polycarbonate filter, Corning, Beijing China), while the normal BMMSC was placed on the lower chamber. The medium was additionally treated with or without GW4869 (10 μM, Sigma-Aldrich) for 24 hr during co-culture with BMMSC. At the end of the experiment, BMMSC were washed with PBS and *cel*-miR-39 expression in BMMSC was detected by qPCR.

### Small RNA (sRNA) sequencing and analysis

Total RNA was extracted from K562 cells and exosomes. The preparation of sRNA libraries and deep sequencing were performed as follows. Briefly, about 500 ng purified RNA from each group was used to prepare the sRNA libraries with Small RNA Sample Pre Kit (Illumina, San Diego, CA, USA). Then miRNA expression was analyzed by small RNA sequence using HiSeq 2500 (Illumina). The differential miRNAs expression was shown by heatmap performed using Cluster3.0 and Java Treeview. Multiple alignment of the mature miRNA sequence of cells and exosomes was performed with ClustalX2 and FigTree software.

An unbiased search to identify over-represented motifs on exosome-enriched miRNAs was performed with Improbizer as previous described 25. Zero-or-one occurrence model was used to find over-represented 4-8 nucleotide-long motifs. The remaining miRNAs annotated in miRBase V22 were used as background. A Markov model of order 0 was assumed for the background sequences. The cladogram was generated with the Weblogo software (http://weblogo.threeplusone.com/).

### *In vitro* osteogenic differentiation assay

Osteogenic differentiation was assayed as previously described 26. Briefly, pre-confluent BMMSC were supplemented with osteogenic medium, α-MEM containing 100 nM dexamethasone (Sigma-Aldrich), 50 µg/mL L-ascorbic acid 2-phosphate, and 10 mM β-glycerophosphate (Sigma-Aldrich), plus either 5% human platelet lysate (HELIOS) for human BMMSC or 10% FBS for mouse BMMSC, for 28 days. After differentiation, cells were fixed with 4% PFA and stained with Alizarin red S (Sigma-Aldrich). For mineralization quantification, destaining was conducted by adding a 10% cetylpyridinium chloride (Sigma-Aldrich) solution. Absorbance was measured in a 96-well plate reader at 570 nm. For alkaline phosphatase (ALP) staining, cells were induced with osteogenic medium for 14 days and ALP activity was measured using BCIP/NBT Alkaline Phosphatase Color Development Kit (Sangon, Shanghai, China) according to manufacturer's instructions.

### Transmission electron microscopy

Exosomes were fixed with 2% paraformaldehyde, loaded on 200-mesh Formvar-coated grids, and then counterstained and embedded as previously described 27. Grids were dried and visualized on a transmission electron microscope at 80 kV.

### RNA immunoprecipitation

RNA immunoprecipitation was performed with cytoplasmic extracts. Briefly, K562 cells or freshly isolated bone marrow mononuclear cells derived from BCR/ABL+ mice were lysed in ice cold lysis buffer (20 mM Tris [pH7.5], 150 mM NaCl, 1% Triton X-100 2 mM sodium pyrophosphate, 25 mM β-glycerophosphate) supplemented with Protease Inhibitor Cocktail (Roche) and 40 U/ml Protector RNase Inhibitors (Roche) for 20 min. The lysates were then centrifuged for 15 min at 12,000g and supernatant was collected. About 1 mg protein extract was incubated with 10 μg mouse anti-HNRNPA1 monoclonal antibody (9H10, Abcam) or 10 μg mouse immunoglobulin G [IgG] (Abcam) for 12 hr at 4 ^o^C at a vertical shaking table. After that, 30 μl protein A sepharose (Abcam) was added for another 2 hr, followed by three washes with ice cold lysis buffer. Co-immunoprecipitated RNA was extracted using Tripure Isolation Reagent (Roche). miRNA qPCR analysis was performed with SYBR green mix (Roche) after reverse transcription using miRcute kit (Tiangen). The miRNA fold enrichment in immunoprecipitated samples was expressed as percentage against input and compared to IgG control.

### Biotinylated miRNA Pull-Down

Biotinylated miRNA pull-down was performed with cytoplasmic extracts. Briefly, 20 nmol biotinylated single stand miRNA oligonucleotides (GenePharma, Shanghai, China) were re-suspended in 100 μL lysis buffer supplemented with Protease Inhibitor Cocktail (Roche) and 40 U/ml Protector RNase Inhibitors (Roche) and incubated with 400 μL protein extract (about 1mg) for 4 hr at 4 ^o^C on a vertical shaking table. Streptavidin sepharose beads (Cell Signaling Technology), pre-washed three times with lysis buffer, were added to the mixture and incubated for an additional 2 hr at 4 ^o^C. The beads were then washed three times with 1 mL lysis buffer each. Beads were mixed with protein loading buffer and heated at 95 ^o^C for 10 min to allow collection of bound proteins for western blot or mass spectrometry (MS) analysis.

### Statistical analysis

Data were analyzed with GraphPad Prism7 software. Unpaired two-tailed t test was used to compare data for two groups. One-way ANOVA test was used to compare data for more than two groups. Data are presented as the mean ± SEM. Differences were considered significant when the *p* value was < 0.05 (* *p*<0.05, ** *p*<0.01, *** p<0.001).

## Results

### Chronic myelogenous leukemia inhibits osteogenesis of bone marrow mesenchymal stromal cells (BMMSC)

**Figure 1:**
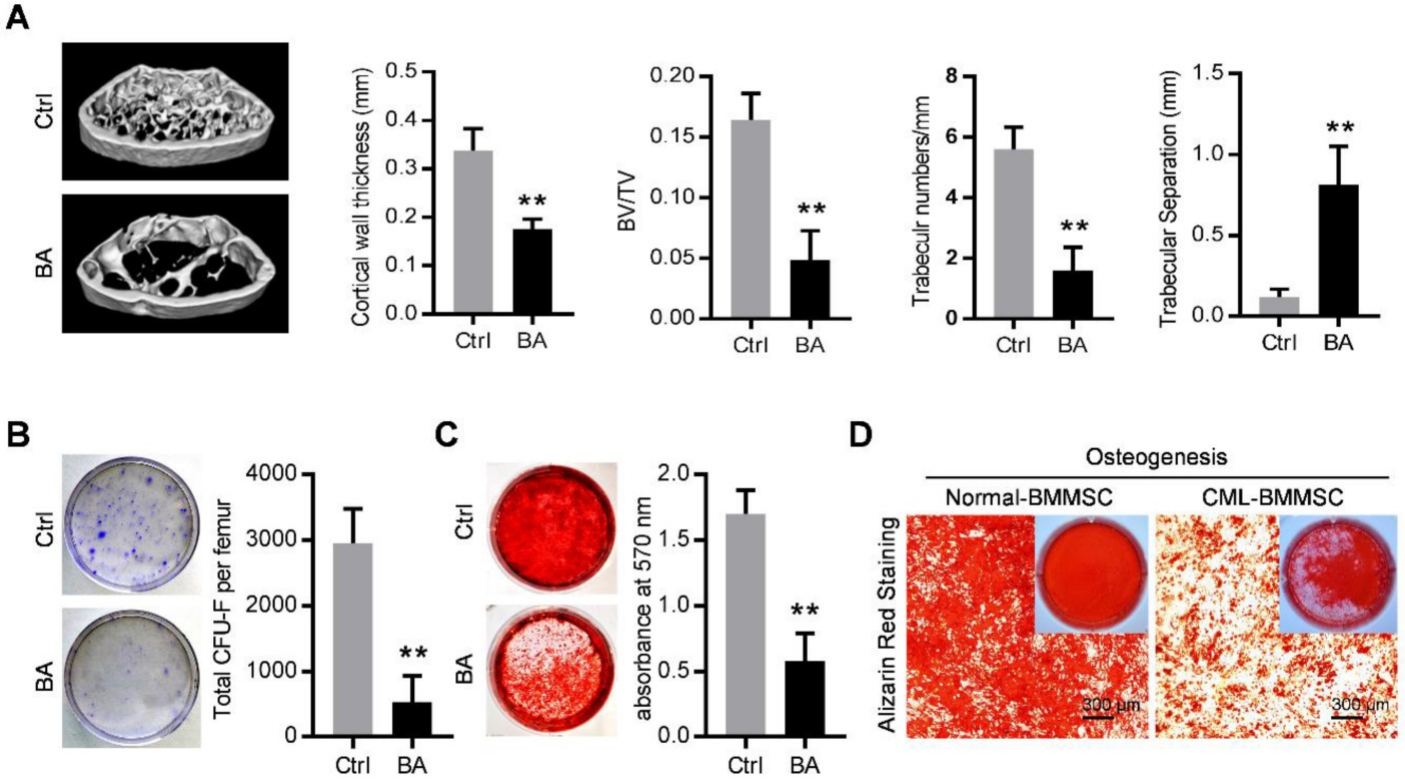
** Functional inhibition of bone marrow mesenchymal stromal cells in chronic myelogenous leukemia.** (A) Representative micro-CT cross-section images of femurs from BCR/ABL+ (BA) mice and control (Ctrl) mice. Bar graphs demonstrate average cortical wall thickness in compact bone regions, relative bone volume (BV/TV), number of trabeculae and space between trabeculae in trabecular bone regions of control and BA mice. Data are expressed as mean ± SEM (n=6 mice per group). (B) Representative images show decreased frequency of CFU-Fs in BMMSC derived from BA mice vs. control mice. Data are expressed as mean ± SEM (n=3 mice per group). (C) Decreased osteogenic differentiation potential of BMMSC from BA mice vs. control mice. Osteogenesis was indicated by alizarin red staining on day 28. Data are expressed as mean ± SEM of three independent experiments. (D) Alizarin red staining photomicrographs of decreased osteogenic differentiation potential of BMMSC from CML patient (CML-BMMSC) vs. age-matched healthy control (Normal-BMMSC). Data presented are representative of 3 normal control and CML patients respectively. ** *p*<0.01 by t test.

The SCL-tTA x TRE-BCR/ABL double transgenic (BA) mice have been widely used as a mouse model of chronic phase CML 8, 21, 28. As expected, BCR/ABL expression following doxycycline withdrawal results in accumulation of myeloid progenitors and mature granulocytes in the bone marrow, spleen and peripheral blood (Figure S1A and S1B) and splenomegaly (Figure S1C), within in one month. With the occurrence of the CML, micro-computed tomography (micro-CT) revealed abnormal bone formation in BA mice after one month induction of BCR/ABL expression. Cortical wall thickness, bone volume relative to tissue volume (BV/TV) and trabecular thickness were significantly decreased, together with an increase of trabecular separation (Figure 1A). Consistent with the observed osteoporosis, BMMSC from induced BA mice exhibited a significant decline of total CFU-F activity per femur as compared to controls (Figure 1B). When cultured under osteoinductive condition, BMMSC from BA mice showed reduced capacity for mineralized nodule formation (Figure 1C). Similarly, BMMSC derived from CML patients also showed reduced capacity for osteogenesis (Figure 1D). These data indicate impairment of osteogenic differentiation and proliferation capacity of BMMSC in both CML mouse model and CML patients.

### Exosomes secreted by leukemia cells are efficiently taken up by BMMSC

**Figure 2:**
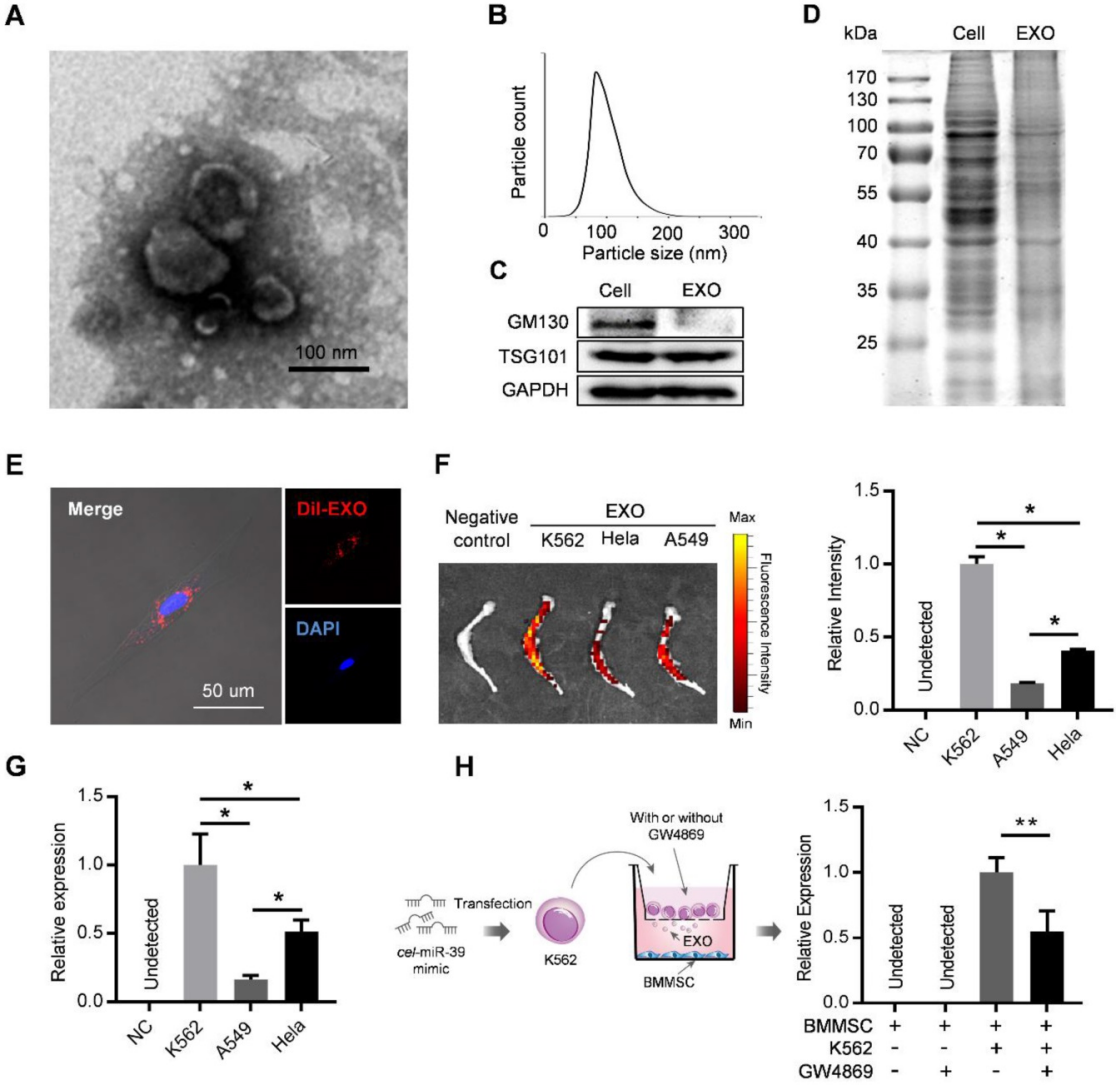
** Leukemia cell-secreted exosomes are efficiently taken up by BMMSC.** (A) Transmission electron microscopy images of exosomes derived from K562 cells. (B) Nanoparticle tracking analysis shows size distribution of K562-derived exosomes. (C) Western blot analysis of exosome-specific marker TSG101 and Golgi-specific marker GM130 in lysates from whole cells (Cell) or exosomes (EXO). GAPDH serves as internal control. (D) Coomassie blue-stained sulfate polyacrylamide gel after electrophoretic separation of total lysates from K562 cells (Cell) and derived exosomes (EXO). (E) Confocal fluorescence image of the endocytosis of DiI-labeled exosomes by primary human BMMSC. (F) *Ex vivo* fluorescence imaging of excised femurs from C57BL/6 mice injected with DiR-labeled exosomes from different cell lines: K562, CML cell line; Hela, human cervical cancer cell line; and A549, human adenocarcinoma cell line. Data are expressed as mean ± SEM (n=3 mice per group). (G) qPCR analysis of cel-miR-39 level in femurs of C57BL/6 mice. Femurs were harvested 24 hours after tail vein injection of exosomes derived from indicated cell lines loaded with cel-miR-39. NC: negative control. Data are expressed as mean ± SEM of three independent experiments. (H) Schematic of co-culture system in Transwell (membrane pore diameter, 0.4 μm) to measure exosome transfer (Left panel). BMMSC were co-cultured with K562 cells transfected with cel-miR-39. The culture medium was added with or without exosome secretion inhibitor GW4869. Cel-miR-39 in BMMSC was quantified by qPCR (right panel). Data are expressed as mean ± SEM of three independent experiments. * *p*<0.05, ** *p*<0.01 by one way ANOVA.

Exosomes derived from cancer cells have been reported to mediate the remodeling of cancer microenvironments, suggesting possible involvement of leukemic exosomes in tuning BMMSC. Exosomes isolated from K562 culture medium by sequential ultracentrifugation exhibited typical cup-shaped morphology by transmission electron microscopy (Figure 2A), with a size distribution of 50 to 150 nm in diameter analyzed by nanoparticle tracking analysis (Figure 2B). Leukemic exosomes were positive for specific exosomal marker TSG101 but negative for GM103, a marker for Golgi complex (Figure 2C). Coomassie blue staining of total protein showed different protein electrophoresis patterns between leukemia cells and leukemic exosomes (Figure 2D). As revealed by confocal fluorescence microscopy, the labeled exosomes were efficiently taken up by BMMSC (Figure 2E). Consistently, *ex vivo* imaging also revealed that these CML-derived exosomes preferentially migrated into the bone marrow, compared with exosomes from other sources, such as cell lines (HeLa and A549) of human solid tumors (Figure 2F). Consistently, intravenous injection of exosomes loaded with a *C. elegans* specific miRNA (*cel*-miR-39) also revealed abundant expression of *cel*-miR-39 in femurs (Figure 2G). To further determine whether leukemic exosomes indeed endocytosed into BMMSC, we transfected K562 cells with *cel*-miR-39, followed by co-culture with BMMSC in a 0.4 µm transwell plate. The appearance of *cel*-miR-39 in BMMSC demonstrates that the *cel*-miR-39 was delivered from the K562 cells in the upper well to the BMMSC in the lower well (Figure 2H). In addition, the exosome secretion inhibitor GW4869 significantly lowered the delivery of *cel*-miR-39 to BMMSC (Figure 2H), further indicating that K562-derived exosomes are efficiently endocytosed into BMMSC. Next, we examined whether BMMSC are the main recipient cells of the leukemic exosomes in the bone marrow (BM) cells. DiO labeled K562 exosomes were intravenously injected into normal C57BL/6 mice. Internalization of the labeled exosomes into mouse BM cells was demonstrated after 24 hr by FACS. Bone marrow mesenchymal stromal cells (CD45- Ter119- CD31-) exhibited the highest fluorescence intensity than other BM cells, such as endothelial cells (CD45- Ter119- CD31+), granulocyte (Mac1+ Gr1+), B cells (B220+), and T cells (CD3+) (Figure S2A), confirming that leukemic exosomes were more efficiently taken up by BMMSC.

### Exosomes from leukemia cells inhibit BMMSC osteogenesis

**Figure 3:**
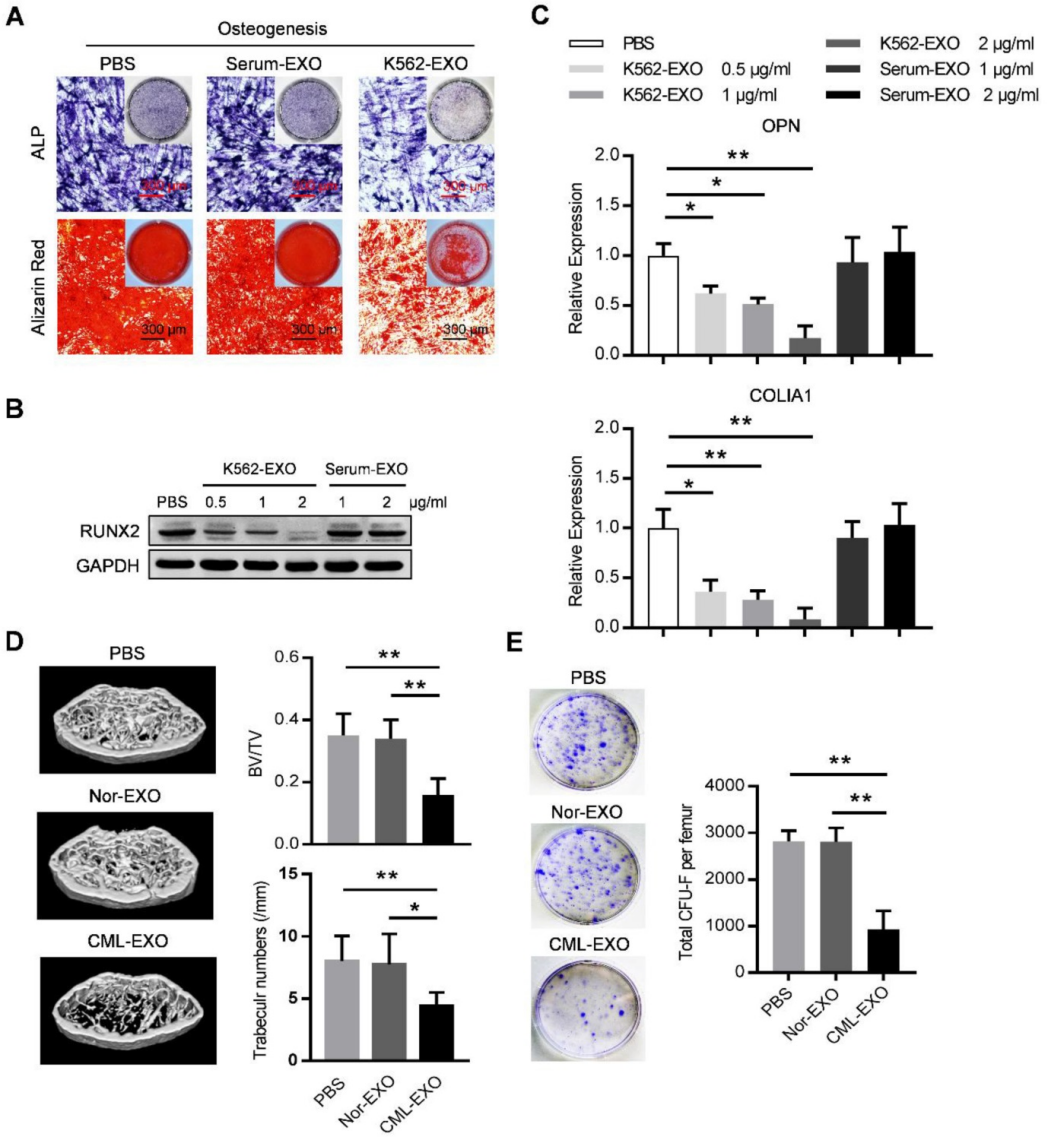
** Leukemia cell-secreted exosomes inhibit BMMSC function *in vitro* and *in vivo*.** (A) ALP and Alizarin red staining analysis of osteogenesis. BMMSC cultured in osteogenic condition were additionally treated with PBS, or exosomes derived from either healthy human serum (Serum-EXO) or cultured K562 cells (K562-EXO) every 3 days, followed by ALP staining after 14 days or by alizarin red staining after 28 days. Images are representative of three independent experiments. (B) Western blot analysis of RUNX2 expression in human BMMSC treated as indicated. Cells were cultured in osteogenic condition for 3 days. Images are representative of three independent experiments. (C) qPCR analysis of mRNA expression of indicated genes in BMMSC treated same as above. Data are presented as the mean ± SEM of three independent experiments. (D) microCT analysis of the femurs from C57BL/6 mice treated as indicated. Mice were intravenously injected with 50 μg of indicated exosomes twice per week for four consecutive weeks (Nor-EXO: exosomes derived from bone marrow mononuclear cells (BM-MNC) of control normal mice; CML-EXO: exosomes derived from BM-MNC of BA mice). Mice treated with PBS served as control. Micro-CT was used to illustrate the effect on cross-sections of femurs in trabecular bone regions and to quantify relative bone volume (BV/TV) and number of trabeculae. Data are expressed as the mean ± SEM (n=6 mice per group). (E) Representative images show the CFU-F of the BMMSC derived from mice with indicated treatments (left panel). Data are presented as the mean ± SEM of three different experiments from 3 mice per group. * *p*<0.05, ** *p*<0.01 by one way ANOVA.

We next explored whether leukemic exosomes can modulate osteogenesis of human BMMSC *in vitro*. BMMSC treated with leukemic exosomes exhibited decreased capacity of osteogenic differentiation as shown by alkaline phosphatase (ALP) and Alizarin Red staining, compared to healthy human serum-derived exosomes or PBS-treated BMMSC (Figure 3A). In addition, the expression of RUNX2, a major osteogenic marker, was downregulated in a dose-dependent manner by treatment with leukemic exosomes (Figure 3B). Furthermore, genes associated with osteogenesis (OPN and COL1A1) or hematopoiesis (CXCL12 and KITL) were also downregulated in BMMSC treated with leukemic exosomes in a dose-dependent manner (Figure 3C and Figure S3A).

Next, we assumed that leukemic exosomes might also inhibit BMMSC functions *in vivo*. C57BL/6 mice (6 to 8 weeks old) were intravenously injected with 50 µg of either leukemic exosomes or control exosomes, derived from BA mice or control mice, twice per week for four consecutive weeks. Mice that received leukemic exosomes exhibited significant loss of trabecular bone and whole bone volume in the femurs (Figure 3D and Figure S3B), similar to the phenotype of transgenic BA mice. Moreover, there was a significant decrease of CFU-F frequencies of BMMSC from leukemic exosome-treated mice, compared to normal controls (Figure 3E). Accordingly, a significant decrease of normal HSC frequency was observed in leukemic exosome-treated mice (Figure S3C), further confirming the niche damage caused by leukemic exosomes. Taken together, these results demonstrate that exosomes secreted by leukemia cells can significantly inhibit the function of BMMSC *in vitro* and *in vivo*.

### Specific repertoires of miRNAs are sorted into leukemia exosomes

**Figure 4:**
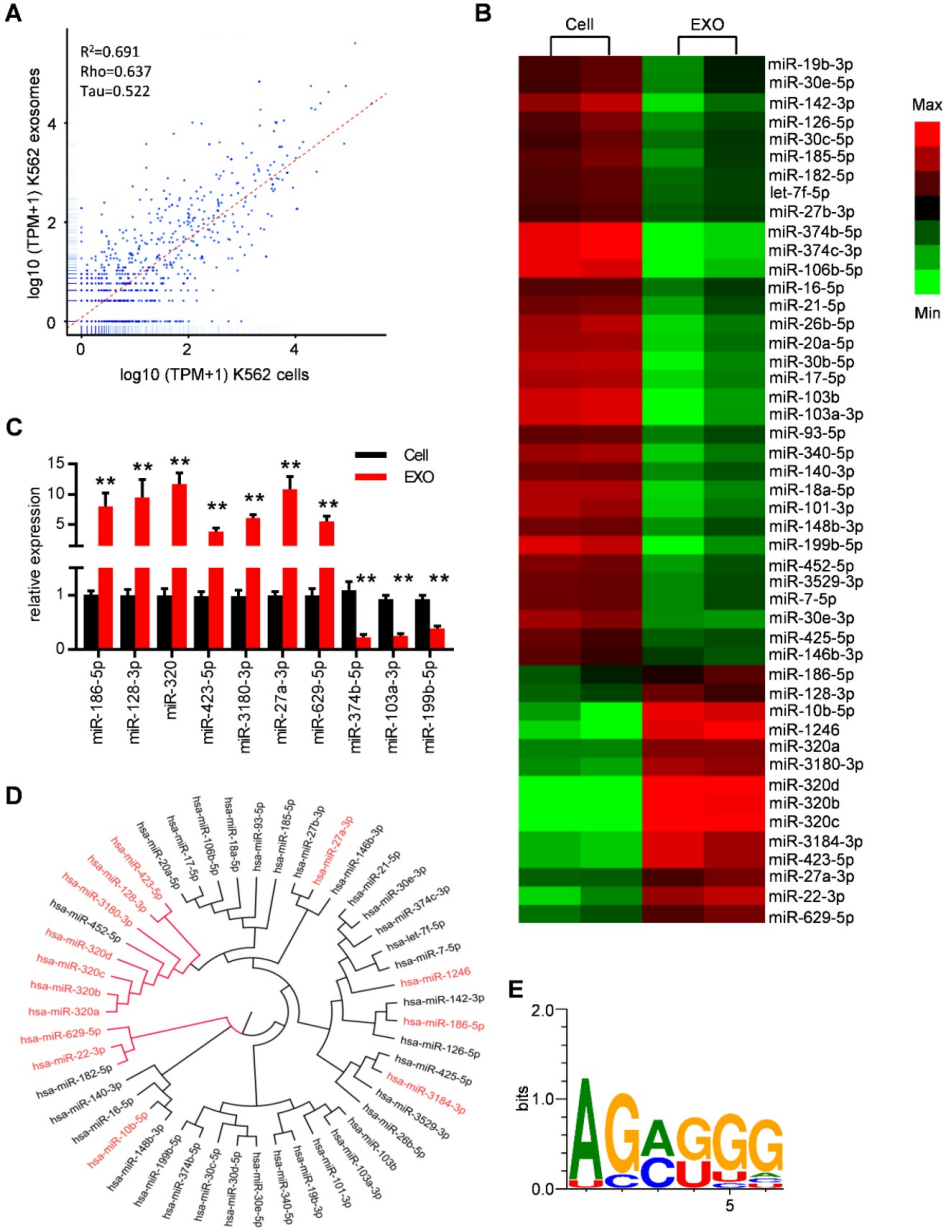
** Specific repertoires of miRNAs are sorted into leukemia exosomes.** (A) Deep sequence data visualization by scatter plot comparing miRNAs in K562 cells and derived exosomes. (B) Heat map depicting differentially expressed miRNAs between cells (Cell) and exosomes (EXO) from human K562 cells. These miRNAs had overall TPM (transcripts per million reads) >1000 in at least one group and fold change difference >2. (C) Verification of selected miRNAs using qPCR. Data are presented as the mean ± SEM of three independent experiments. ** *p*<0.01 by t test. (D) Cladogram showing the multiple alignment of mature sequences of 14 leukemic exosome-enriched miRNAs (red) and 31 cell-enriched miRNAs (black). (E) Over-represented motifs on exosome-enriched miRNAs. The core AGAGGG shows high information content.

MiRNAs have been reported to be key exosome cargo that can be actively secreted into exosomes and delivered to target cells, resulting in direct modulation of their mRNA targets. In order to clarify whether miRNAs are the key functional mediators in the observed function of leukemic exosomes, we thus performed small RNA sequencing and analyzed the intracellular and exosomal profile of the miRNome in K562 cells (Figure 4A and Figure S4A). In particular, 148 miRNAs were enriched and 165 miRNAs were under represented in leukemic exosomes compared to the cellular fraction. These miRNAs were further analyzed based on fold changes and overall TPM (transcripts per million reads) (at least two data >1000). Forty seven of the miRNAs met these criteria and were selected for further analysis (Figure S4B, Table S5). A heat map of the selected miRNAs demonstrated the profound difference between leukemic cells and exosomes (Figure 4B). The expression profile was further confirmed by qPCR analysis of selected miRNAs in both K562 cells (Figure 4C) and LAMA84 cells (Figure S4C). These results showed that distinct subsets of miRNAs were either enriched or diminished in leukemic exosomes.

ClustalW was used to find the sequence characteristics of the enriched/diminished miRNAs. As shown in Figure 4D, a group of miRNAs that were enriched in exosomes harbored a similar mature sequence, including miR-320a, miR-320b, miR-320c, miR-320d, miR-3180-3p, miR-128-3p and miR-423-5p. An unbiased search for over-represented sequence motifs using Improbizer 25 detected a conserved minimal AGAGGG motif (Figure 4E). About 50% of exosome-enriched miRNAs contain at least 4 nucleotides of the motif but only 9.7% of cell-enriched miRNAs (Table S5). These data indicated that specific repertoires of miRNAs with certain motifs were sorted into leukemia exosomes.

### Exosomal miR-320 plays an essential role in the observed effects on both leukemia cells and BMMSC

**Figure 5:**
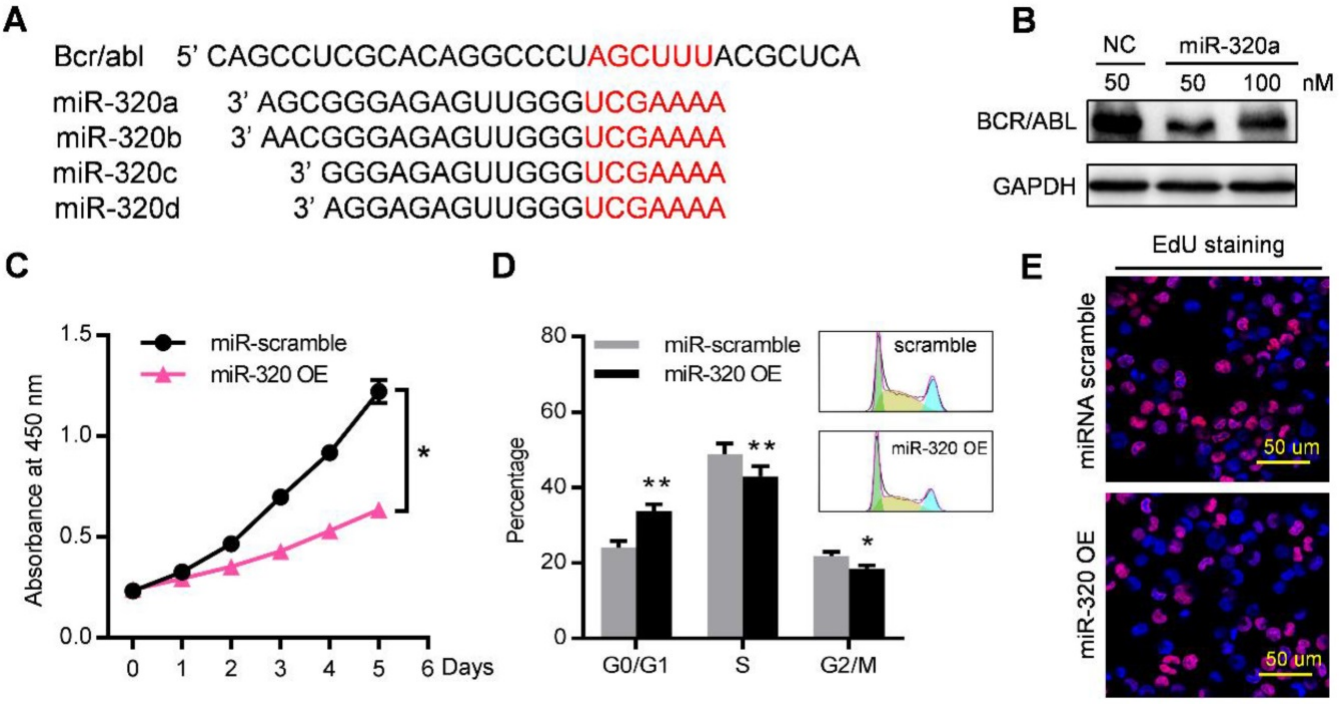
** miR-320 inhibits CML proliferation by directly targeting BCR/ABL.** (A) Schematic diagram of the putative binding sites of the miR-320 family in the 3' untranslated region (UTR) of bcr/abl. (B) BCR/ABL (P210) protein level, measured by western blot, in K562 cells transfected with different dose of miR-320a mimics or scramble (NC) for 48 hr. Images are representative of three independent experiments. (C) Proliferation of K562 cells with miR-320a (miR-320 OE) or scrambled miRNA (miR-scramble) overexpression, as analyzed by CCK-8 assay. (D) Cell cycle phase analysis of K562 cells transfected with either miR-320a (miR-320 OE) or scrambled control (miR-scramble). The percentage of cells in the G1, S, and G2 phases is shown in the bar graph. Data are presented as the mean ± SEM of three independent experiments. (E) EdU incorporation assay of K562 transfected with either miR-320a (miR-320 OE) or scrambled miRNA (miR-scramble). Images are representative of three independent experiments. * *p*<0.05, ** *p*<0.01 by t test or two way ANOVA.

Among these miRNAs that were enriched in leukemic exosomes, we focused on the miR-320 family for three reasons: (1) family members such as miR-320a, miR-320b, miR-320c and miR-320d were specifically loaded into leukemic exosomes (Figure 4B, 4D, S4B); (2) miR-320 family contains the AGAGGG motif which may control their exosomal sorting; and (3) previous research has shown that miR-320 can directly target the BCR/ABL oncogene 29, and β-catenin 30, 31 which is a key regulator of osteogenesis. We then investigated the functional effect of miR-320 on both leukemic cells and BMMSC. The 3'-UTR of BCR/ABL contains a binding site that can be recognized by all four miR-320 as predicted from a miRNA target database (Figure 5A). As expected, transfection of miR-320a mimics significantly decreased the endogenous expression of BCR/ABL in K562 cells (Figure 5B). Forced expression of miR-320a has significantly inhibited K562 cell proliferation (Figure 5C). Cell cycle assessment suggested that miR-320a can cause G1 phase arrest in the cell cycle (Figure 5D), which was further confirmed by EdU incorporation assay (Figure 5E).

**Figure 6:**
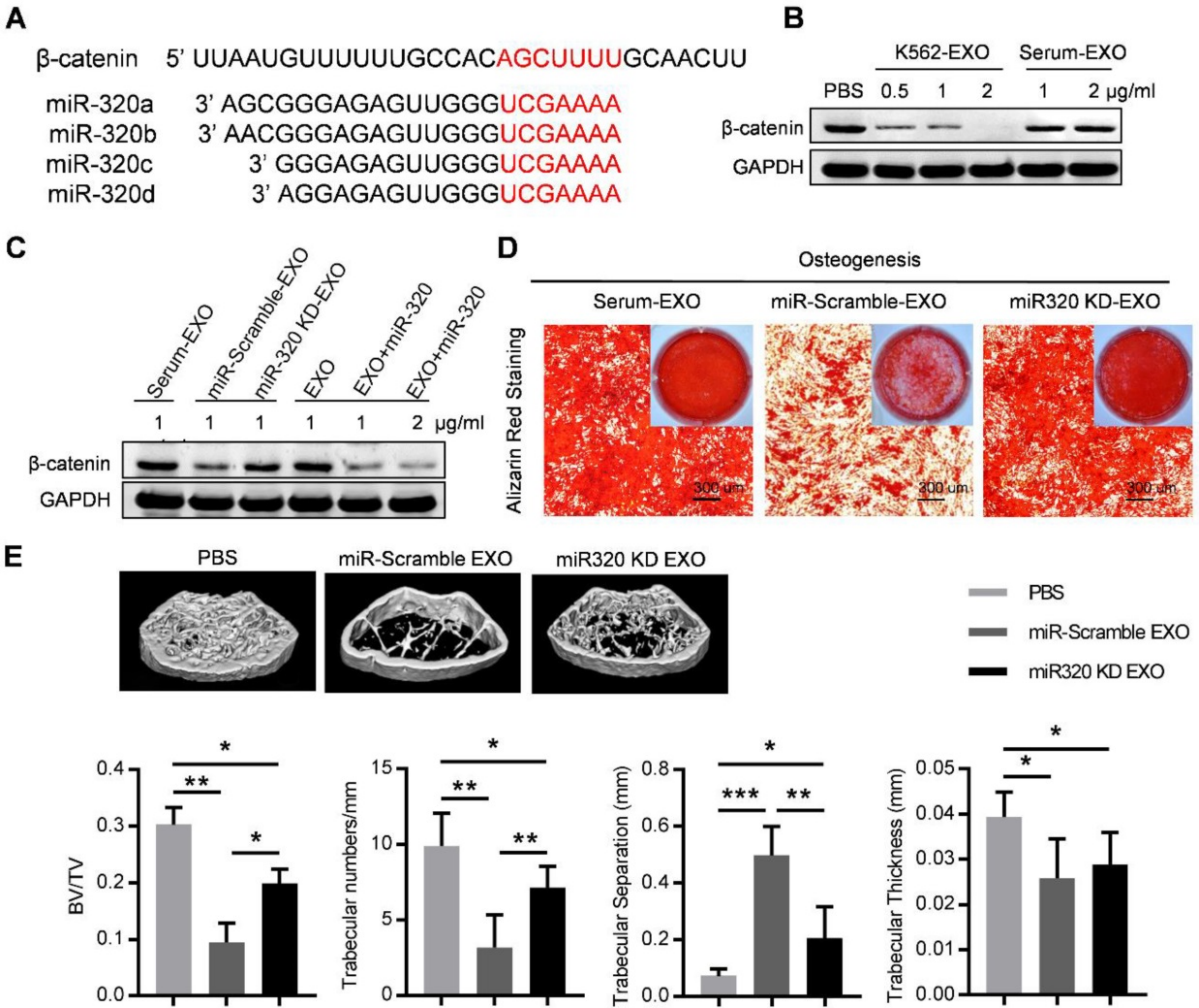
** Exosomal miR-320 inhibits BMMSC osteogenesis.** (A) Schematic diagram of the putative binding sites of the miR-320 family in the 3'UTR of β-catenin. (B) Expression of β-catenin in BMMSC with indicated treatments. Images are representative of three independent experiments. (C) Expression of β-catenin in BMMSC with indicated exosome treatments. Serum-EXO: healthy human serum exosomes; miR-Scramble-EXO and miR-320 KD-EXO: exosomes from control or miR-320 knocked down K562 cells (infected with scramble or miR-320-targerted sponge sequence vector); EXO and EXO+miR-320: exosomes from HEK293T cells loaded without or with miR-320. Images are representative of three independent experiments. (D) Alizarin red staining showing the osteogenic differentiation of BMMSC treated with indicated exosomes. BMMSC treated with healthy human serum exosomes (Serum-EXO) served as control. Images are representative of 3 independent experiments. (E) Micro-CT analysis of the femurs of mice treated as indicated. C57BL/6 mice were tail vein injected with 50 μg of indicated exosomes twice per week for four consecutive weeks. Mice treated with PBS served as control. Relative bone volume (BV/TV), trabecular numbers, trabecular separation, and trabecular thickness, were further analyzed. Data are presented as the mean ± SEM (n=6 mice per group). * *p*<0.05, ** *p*<0.01, *** *p*<0.001 by one way ANOVA.

Notably, the 3'-UTR of β-catenin also contains a binding site that can be recognized by all four miR-320 (Figure 6A). β-catenin is the transcriptional regulator of canonical Wnt signaling, which can promote the maturation of osteoblastic precursor cells into mature osteoblasts. Knocking down of β-catenin in BMMSC by siRNAs inhibited the osteogensis as measured by ALP staining (Figure S5A), and reduced the expression of osteogenesis (OPN, COLIA1) or hematopoiesis (CXCL12, KITL) related genes (Figure S5B). K562 derived exosomes significantly decreased the expression of β-catenin in BMMSC, which was rescued by prior miR-320 knockdown in K562 cells (Figure 6B and 6C). Accordingly, the inhibited osteogenic differentiation capacity of BMMSC by exosomes derived from K562 cells was compromised when miR-320a was knocked down in the parental cells (Figure 6D). To further confirm the role of aberrant differentiation of BMMSC induced by miR-320, we have knocked down miR-320 (miR-320 KD) in K562 cells, and then injected 50 µg of the derived exosomes into the C57BL/6 mice, twice per week for four consecutive weeks. Compared with the control groups, exosomes from miR-320 KD K562 cells had minimal effects on the bone abnormalities (Figure 6E). These data indicate that miR-320 is essential for the observed effects of K562 exosomes on both K562 cells and BMMSC.

### HNRNPA1 mediates exosomal sorting of miR-320

To unravel the molecular mechanisms that control the active sorting into exosomes of specific miRNAs, especially the miR-320 family, K562 cell lysates were incubated with streptavidin beads coated with biotinylated miR-320a. The bound proteins were subjected to high-throughput identification by mass spectrometry (MS). Non-coated beads and beads with biotinylated miR-374b-5p (which was a cell-enriched miRNA) served as negative control. Among the enriched proteins precipitated with biotinylated miRNA-320a (Table S4), we focused on HNRNPA1, a ubiquitously expressed RNA-binding protein recognizing the motif UAGGG(A/U) (Figure S6A).

**Figure 7:**
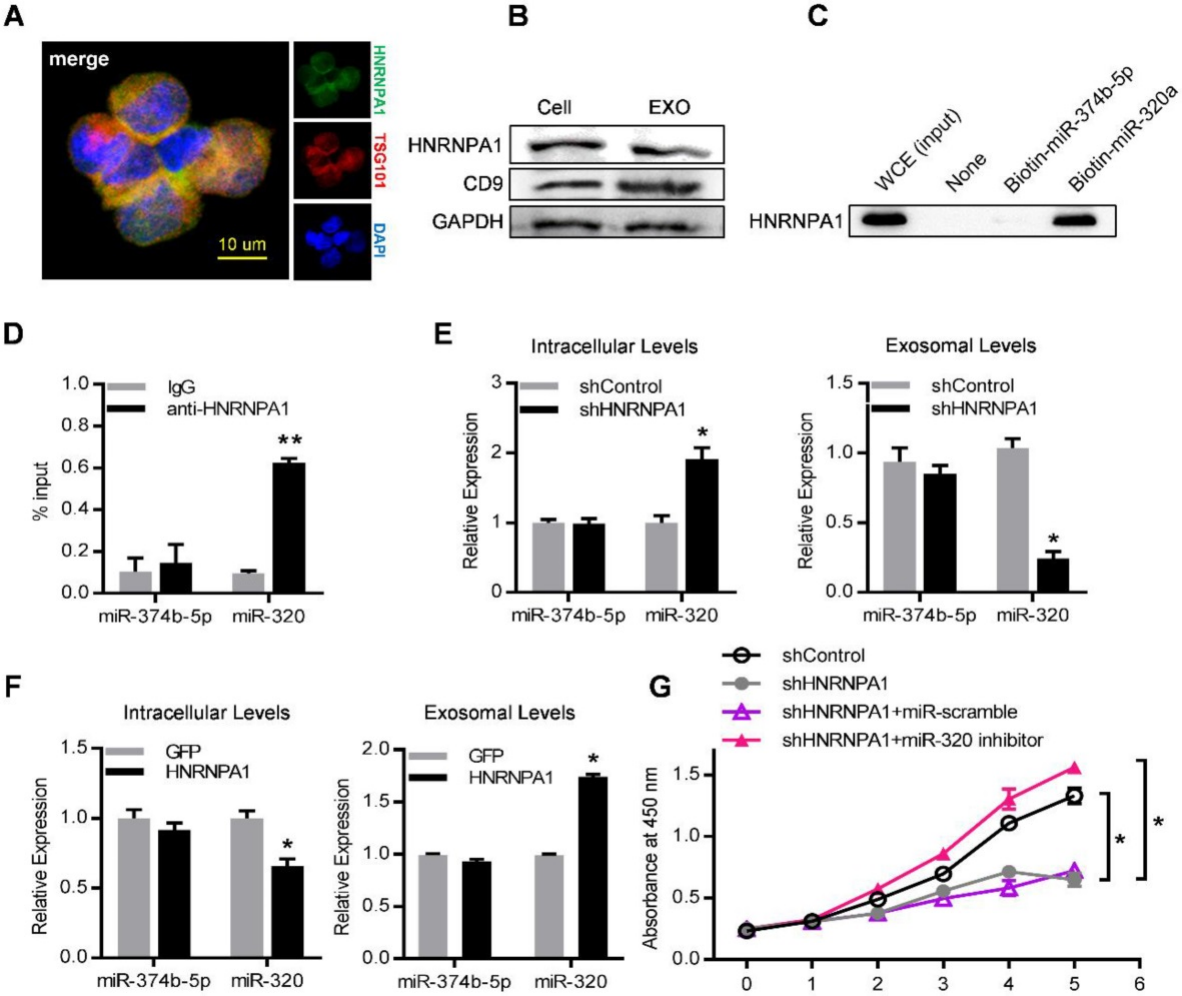
** HNRNPA1 mediates exosomal sorting of miR-320.** (A) Confocal microscopy image shows the co-localization of HNRNPA1 (green) and specific exosome marker TSG101 (red) in K562 cells. (B) Western blot showing HNRNPA1 and CD9 expression in both cellular (Cell) and exosomal (EXO) lysates. GAPDH serves as internal control. (C) Western blot showing HNRNPA1 pulled down by biotinylated miR-320 in whole cellular extracts (WCE) of K562 cells, with biotinylated miR-374b-5p serving as control. Images are representative of three independent experiments. (D) RNA-IP analysis of miR-320 bound by HNRNPA1. K562 cytoplasmic extracts were incubated with IgG or antiHNRNPA1 and the immunoprecipates were isolated for qRT-PCR analysis. miR-374b-5p serves as a negative control. Data are means ± SEM of three independent experiments. (E) qRT-PCR analysis of intracellular (left) and exosomal (right) levels of selected miRNAs in HNRNPA1 knock down (shHNRNPA1) or control K562 cells (shControl). The values were normalized to U6 levels in cells or exosomes and shown as mean ± SEM of three independent experiments. (F) qRT-PCR analysis of intracellular (left) and exosomal (right) levels of selected miRNAs in control (GFP) or HNRNPA1 overexpression K562 cells. Data are means ± SEM of three independent experiments. (G) Proliferation of K562 cells treated as indicated were further analyzed by CCK-8 assay. Data are expressed as means ± SEM of 3 different experiments. * p<0.05, ** *p*<0.01, *** *p*<0.001 by t test.

Consistently, HNRNPA1 was abundantly expressed in both leukemic cells and their exosomes, as confirmed by both immunofluorescence confocal microscopy (Figure 7A) and western blot (Figure 7B). Specific binding of HNRNPA1 to miR-320a was verified with miRNA pull-down followed by western blot analysis of HNRNPA1 (Figure 7C). HNRNPA1 can be efficiently pulled down by biotinylated miR-320a, in comparison to biotinylated miR-374b-5p, demonstrating specific binding of HNRNPA1 and miR-320a in cells. The result was further confirmed with immunoprecipitation of HNRNPA1 from cytoplasmic lysates of both K562 cells (Figure 7D) and bone marrow mononuclear cells from BA mice (Figure S6B). The effects of HNRNPA1 knockdown and overexpression (Figure S6C) on miR-320 sorting into exosomes were further assessed. After HNRNPA1 knockdown, miR-320 was significantly increased in the cellular components but decreased in the exosomes (Figure 7E). In contrast, overexpression of HNRNPA1 increased the exosomal enrichment but decreased the intracellular abundance of miR-320 (Figure 7F). No significant change of cellular and exosomal expression of cell-enriched miR-374b-5p was found upon manipulating HNRNPA1 expression. Consistent with the role of HNRNPA1 in exosomal sorting of miR-320, knockdown of HNRNPA1 significantly decreased the growth rate of K562 cells, which can be rescued by miR-320 inhibitor (Figure 7G). Notably, inhibition of BCR/ABL by Imatinib decreased both the cellular and exosomal HNRNPA1 protein level (Figure S6D), together with increased miR-320 in the cells while decreased level in the exosomes (Figure S6E). Collectively, these data demonstrate that HNRNPA1 functions importantly in the sorting of miR-320 into leukemic exosomes, and Imatinib might inhibit leukemia cell growth partially via interfering the pathway.

### Abnormal expression of HNRNPA1 and exosomal miR-320 in clinical CML samples

**Figure 8:**
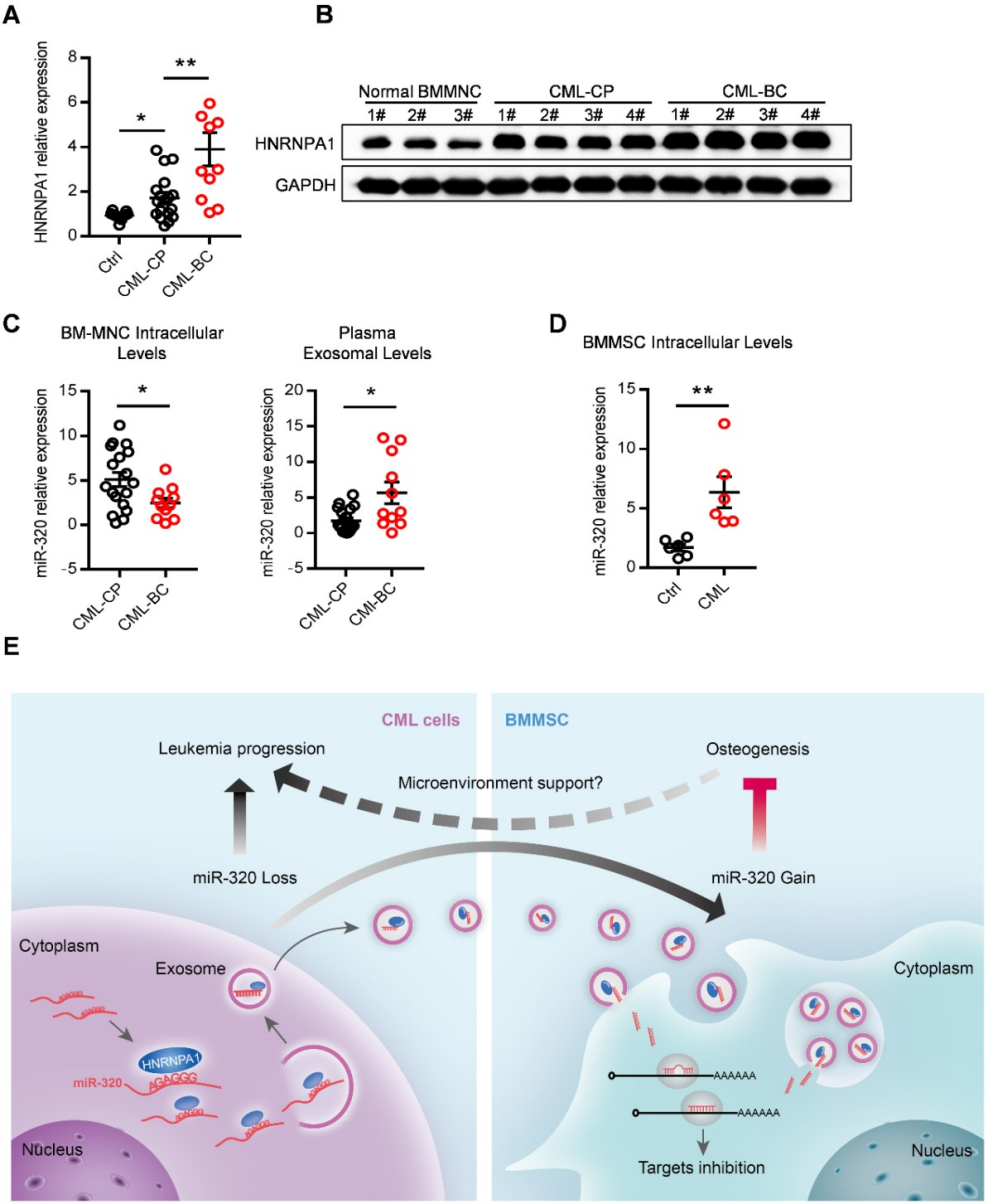
** Abnormal expression of HNRNPA1 and miR-320 in chronic myelogenous leukemia patients.** mRNA (A) and protein (B) expression of HNRNPA1 in primary human bone marrow mononuclear cells (BM-MNC) of normal control, chronic phase (CML-CP) or blast crisis CML (CML-BC) patients. (C) BM-MNC cellular and plasma exosomal expression of miR-320 in CML-CP or CML-BC patients. (D) Intracellular expression level of miR-320 in BMMSC derived from healthy control and CML patients. (E) Graphic illustration of the study: HNRNPA1 mediated exosomal transfer of miR-320 from leukemia cell to mesenchymal stromal cell simultaneously promotes leukemia cell growth while inhibits mesenchymal stromal cell osteogenesis. * *p*<0.05, ** *p*<0.01 by t test or one way ANOVA.

Finally, we asked whether our findings in the leukemic cell line and murine model had clinical relevance. HNRNPA1 mRNA and protein levels were markedly increased in chronic phase CML (CML-CP) cells and became even higher in blast crisis CML (CML-BC) cells (Figure 8A and 8B), compared to normal BM cells. Moreover, CML-BC patients had a much lower miR-320 expression in intracellular levels but higher in plasma exosomal levels, compared to CML-CP patients (Figure 8C), which might be explained by the higher expression of HNRNPA1 in CML-BC cells. In addition, some other differentially miRNAs identified in the RNA-seq data were also found expressed in a similar pattern in the CML cells and plasma exosomes (Figure S7A). Consistent with the assumption that exosomal miR-320 might be transported from leukemic cells to BMMSC, there were higher intracellular level of miR-320 in primary BMMSC derived from CML patients than from healthy donors (Figure 8E).

## Discussion

In this study, we have found that the exosomes secreted from leukemia cells reduced the tumor suppressive miR-320 in the donor cells, resulting promoted cell growth. Moreover, we found that leukemic exosomes can decrease the osteogenic differentiation potential of BMMSC both *in vitro* and in mouse model, resulting in reduced trabecular bone volume. The study demonstrate that the exosome derived from leukemia cells is an important contributor of niche remodeling.

The functional importance of bone marrow niche remodeling for failure of normal hematopoiesis in myeloproliferative neoplasms has become increasingly evident 5, 7, 8, 32. During the progress of myeloproliferative neoplasms, the functions of bone marrow mesenchymal stromal cells (BMMSC) are profoundly impaired. AML-derived BMMSCs are molecularly and functionally altered with decreased proliferation and osteogenic differentiation, which contribute to hematopoietic insufficiency 2, 3, 33-35. The mesenchymal niche of CML is also found aggressively modified into a self-reinforcing leukemic niche that impairs normal hematopoiesis 5, 7, 32. Previous studies have suggested an essential role for tumor cell-derived exosomes in reprogramming the tumor microenvironment. Kumar et al 36 found that acute myeloid leukemia cell-derived exosomes can transform the bone marrow niche into a leukemia-permissive microenvironment by impairing the normal functions of BMMSC. Here, we further revealed that CML secreted exosomes could simultaneously promote the CML cell growth and remodel the bone marrow niche toward leukemia permissive microenvironment via inhibiting osteogenesis.

Exosomes derived from CML cells can shuttle miRNAs, amphiregulin and even BCR/ABL mRNAs to nearby stromal cells 37-39, resulting in reprogramming of niche cell functions. In our study, deep sequencing of small RNAs in leukemic cells and their exosomes shows a profound differences between intracellular and exosomal miRNAs. Among the exosome-enriched miRNAs, a higher proportion has previously been identified as tumor suppressive miRNAs 29, 30, 40, 41, including miR-320 family, miR-3180-3p, miR-128-3p and miR-423-5p, compared to cell-enriched miRNAs. Based on our observation, leukemia cells are more likely to selectively export these suppressive miRNAs to promote tumor cell growth. Although we here focused on the role of tumor suppressor miR-320, the function of other miRNAs should not be excluded. In fact, all of the encapsulated miRNAs might work together to remodel the niche, while secretion of these miRNAs via exosomes also promotes the donor cell proliferation.

To date, different pathways and molecules have been described to involved in the sorting of specific miRNAs into exosomes. For example, heterogeneous nuclear ribonucleoprotein A2B1 (HNRNPA2B1) and heterogeneous nuclear ribonucleoprotein Q (HNRNPQ) can bind to miRNAs containing a certain sequence (called EXO motif) and guide their inclusion into exosomes 17, 18. In addition, major vault protein (MVP) is also reported to mediate the loading of miRNAs into exosomes 42. We here found that HNRNPA1 at least selectively sorted out miR-320 into exosomes via the AGAGGG motif in the miRNAs. HNRNPA1 is a highly abundant RNA-binding protein that has been implicated in diverse cellular functions related to RNA metabolism including nucleo-cytoplasmic shuttling 43, alternative splicing regulation 44, mRNA stability and miRNA processing 45. Indeed, some leukemia-related factors such as BCL-XL and SET are processed by HNRNPA1 20. HNRNPA1 expression is also induced by BCR/ABL protein in a dose and kinase-dependent manner 20. Consistent with previous research, HNRNPA1 levels were higher in CML-CP cells, and even higher in CML-BC cells, compared to normal bone marrow cells. Together, our findings suggest a novel mechanism of how HNRNPA1 promotes leukemia progression: by selectively sorting tumor suppressor miRNAs into exosomes. As leukemia stem cells (LSC) are the origin of CML, it is thus worthwhile to confirm whether the HNRNPA1 mediated miR-320 sorting into exosomes occurs in LSCs. It is also important to compare the differences between HSC and LSC derived exosomes, while the study is limited by the technical difficulty of *in vitro* culture of HSCs and LSCs without feeder cells. Single cell sequencing might be the solution.

In summary, this study has revealed that HNRNPA1 promotes cell growth via exosomal sorting of tumor suppressive miRNAs (mainly miR-320) in CML, while the secreted exosomes in turn function to remodel the niche to a leukemic favorable microenvironment via inhibiting osteogenesis of BMMSC (Figure 8E). Thus, HNRNPA1 and the exosomal miRNAs provide novel therapeutic targets to restore the normal function of the bone marrow niche.

## Supplementary Material

Supplementary figures and tables.

## References

[1] Schepers K, Campbell TB, Passegue E (2015). Normal and leukemic stem cell niches: insights and therapeutic opportunities. Cell Stem Cell.

[2] Geyh S, Rodriguez-Paredes M, Jager P, Khandanpour C, Cadeddu RP, Gutekunst J (2016). Functional inhibition of mesenchymal stromal cells in acute myeloid leukemia. Leukemia.

[3] von der Heide EK, Neumann M, Vosberg S, James AR, Schroeder MP, Ortiz-Tanchez J (2017). Molecular alterations in bone marrow mesenchymal stromal cells derived from acute myeloid leukemia patients. Leukemia.

[4] Kim JA, Shim JS, Lee GY, Yim HW, Kim TM, Kim M (2015). Microenvironmental remodeling as a parameter and prognostic factor of heterogeneous leukemogenesis in acute myelogenous leukemia. Cancer Res.

[5] Schepers K, Pietras EM, Reynaud D, Flach J, Binnewies M, Garg T (2013). Myeloproliferative neoplasia remodels the endosteal bone marrow niche into a self-reinforcing leukemic niche. Cell Stem Cell.

[6] Colmone A, Amorim M, Pontier AL, Wang S, Jablonski E, Sipkins DA (2008). Leukemic cells create bone marrow niches that disrupt the behavior of normal hematopoietic progenitor cells. Science.

[7] Zhang B, Ho YW, Huang Q, Maeda T, Lin A, Lee SU (2012). Altered microenvironmental regulation of leukemic and normal stem cells in chronic myelogenous leukemia. Cancer Cell.

[8] Welner RS, Amabile G, Bararia D, Czibere A, Yang H, Zhang H (2015). Treatment of chronic myelogenous leukemia by blocking cytokine alterations found in normal stem and progenitor cells. Cancer Cell.

[9] Tkach M, Thery C (2016). Communication by Extracellular Vesicles: Where we are and where we need to go. Cell.

[10] van Niel G, D'Angelo G, Raposo G (2018). Shedding light on the cell biology of extracellular vesicles. Nat Rev Mol Cell Biol.

[11] Zhou W, Fong MY, Min Y, Somlo G, Liu L, Palomares MR (2014). Cancer-secreted miR-105 destroys vascular endothelial barriers to promote metastasis. Cancer Cell.

[12] Bruns H, Bottcher M, Qorraj M, Fabri M, Jitschin S, Dindorf J (2017). CLL-cell-mediated MDSC induction by exosomal miR-155 transfer is disrupted by vitamin D. Leukemia.

[13] Zhang L, Zhang S, Yao J, Lowery FJ, Zhang Q, Huang WC (2015). Microenvironment-induced PTEN loss by exosomal microRNA primes brain metastasis outgrowth. Nature.

[14] Hornick NI, Doron B, Abdelhamed S, Huan J, Harrington CA, Shen R (2016). AML suppresses hematopoiesis by releasing exosomes that contain microRNAs targeting c-MYB. Sci Signal.

[15] Boyiadzis M, Whiteside TL (2017). The emerging roles of tumor-derived exosomes in hematological malignancies. Leukemia.

[16] Zietzer A, Werner N, Jansen F (2018). Regulatory mechanisms of microRNA sorting into extracellular vesicles. Acta Physiol (Oxf).

[17] Villarroya-Beltri C, Gutierrez-Vazquez C, Sanchez-Cabo F, Perez-Hernandez D, Vazquez J, Martin-Cofreces N (2013). Sumoylated hnRNPA2B1 controls the sorting of miRNAs into exosomes through binding to specific motifs. Nat Commun.

[18] Santangelo L, Giurato G, Cicchini C, Montaldo C, Mancone C, Tarallo R (2016). The RNA-binding protein SYNCRIP is a component of the hepatocyte exosomal machinery controlling microRNA sorting. Cell Rep.

[19] Perrotti D, Neviani P (2007). From mRNA metabolism to cancer therapy: chronic myelogenous leukemia shows the way. Clin Cancer Res.

[20] Iervolino A, Santilli G, Trotta R, Guerzoni C, Cesi V, Bergamaschi A (2002). hnRNP A1 nucleocytoplasmic shuttling activity is required for normal myelopoiesis and BCR/ABL leukemogenesis. Mol Cell Biol.

[21] Koschmieder S, Gottgens B, Zhang P, Iwasaki-Arai J, Akashi K, Kutok JL (2005). Inducible chronic phase of myeloid leukemia with expansion of hematopoietic stem cells in a transgenic model of BCR-ABL leukemogenesis. Blood.

[22] Studeny M, Marini FC, Dembinski JL, Zompetta C, Cabreira-Hansen M, Bekele BN (2004). Mesenchymal stem cells: potential precursors for tumor stroma and targeted-delivery vehicles for anticancer agents. J Natl Cancer Inst.

[23] Abbuehl JP, Tatarova Z, Held W, Huelsken J (2017). Long-term engraftment of primary bone marrow stromal cells repairs niche damage and improves hematopoietic stem cell transplantation. Cell Stem Cell.

[24] Schmittgen TD, Livak KJ (2008). Analyzing real-time PCR data by the comparative CT method. Nat Protoc.

[25] Ao W, Gaudet J, Kent WJ, Muttumu S, Mango SE (2004). Environmentally induced foregut remodeling by PHA-4/FoxA and DAF-12/NHR. Science.

[26] Ambrosi TH, Scialdone A, Graja A, Gohlke S, Jank AM, Bocian C (2017). Adipocyte accumulation in the bone marrow during obesity and aging impairs stem cell-based hematopoietic and bone regeneration. Cell Stem Cell.

[27] Wang J, Hendrix A, Hernot S, Lemaire M, De Bruyne E, Van Valckenborgh E (2014). Bone marrow stromal cell-derived exosomes as communicators in drug resistance in multiple myeloma cells. Blood.

[28] Reynaud D, Pietras E, Barry-Holson K, Mir A, Binnewies M, Jeanne M (2011). IL-6 controls leukemic multipotent progenitor cell fate and contributes to chronic myelogenous leukemia development. Cancer Cell.

[29] Xishan Z, Ziying L, Jing D, Gang L (2015). MicroRNA-320a acts as a tumor suppressor by targeting BCR/ABL oncogene in chronic myeloid leukemia. Sci Rep.

[30] Yuan SX, Wang J, Yang F, Tao QF, Zhang J, Wang LL (2016). Long noncoding RNA DANCR increases stemness features of hepatocellular carcinoma by derepression of CTNNB1. Hepatology.

[31] Kang DW, Noh YN, Hwang WC, Choi KY, Min do S (2016). Rebamipide attenuates Helicobacter pylori CagA-induced self-renewal capacity via modulation of beta-catenin signaling axis in gastric cancer-initiating cells. Biochem Pharmacol.

[32] Arranz L, Sanchez-Aguilera A, Martin-Perez D, Isern J, Langa X, Tzankov A (2014). Neuropathy of haematopoietic stem cell niche is essential for myeloproliferative neoplasms. Nature.

[33] Huang JC, Basu SK, Zhao X, Chien S, Fang M, Oehler VG (2015). Mesenchymal stromal cells derived from acute myeloid leukemia bone marrow exhibit aberrant cytogenetics and cytokine elaboration. Blood Cancer J.

[34] Jacamo R, Davis RE, Ling X, Sonnylal S, Wang Z, Ma W (2017). Tumor Trp53 status and genotype affect the bone marrow microenvironment in acute myeloid leukemia. Oncotarget.

[35] Kornblau SM, Ruvolo PP, Wang RY, Battula VL, Shpall EJ, Ruvolo VR (2018). Distinct protein signatures of acute myeloid leukemia bone marrow-derived stromal cells are prognostic for patient survival. Haematologica.

[36] Kumar B, Garcia M, Weng L, Jung X, Murakami JL, Hu X (2018). Acute myeloid leukemia transforms the bone marrow niche into a leukemia-permissive microenvironment through exosome secretion. Leukemia.

[37] Ramos TL, Sanchez-Abarca LI, Lopez-Ruano G, Muntion S, Preciado S, Hernandez-Ruano M (2015). Do endothelial cells belong to the primitive stem leukemic clone in CML? Role of extracellular vesicles. Leuk Res.

[38] Corrado C, Saieva L, Raimondo S, Santoro A, De Leo G, Alessandro R (2016). Chronic myelogenous leukaemia exosomes modulate bone marrow microenvironment through activation of epidermal growth factor receptor. J Cell Mol Med.

[39] Taverna S, Amodeo V, Saieva L, Russo A, Giallombardo M, De Leo G (2014). Exosomal shuttling of miR-126 in endothelial cells modulates adhesive and migratory abilities of chronic myelogenous leukemia cells. Mol Cancer.

[40] Aslan D, Garde C, Nygaard MK, Helbo AS, Dimopoulos K, Hansen JW (2016). Tumor suppressor microRNAs are downregulated in myelodysplastic syndrome with spliceosome mutations. Oncotarget.

[41] Yu WW, Jiang H, Zhang CT, Peng Y (2017). The SNAIL/miR-128 axis regulated growth, invasion, metastasis, and epithelial-to-mesenchymal transition of gastric cancer. Oncotarget.

[42] Teng Y, Ren Y, Hu X, Mu J, Samykutty A, Zhuang X (2017). MVP-mediated exosomal sorting of miR-193a promotes colon cancer progression. Nat Commun.

[43] Pinol-Roma S, Dreyfuss G (1992). Shuttling of pre-mRNA binding proteins between nucleus and cytoplasm. Nature.

[44] Tavanez JP, Madl T, Kooshapur H, Sattler M, Valcarcel J (2012). hnRNP A1 proofreads 3' splice site recognition by U2AF. Mol Cell.

[45] Kooshapur H, Choudhury NR, Simon B, Muhlbauer M, Jussupow A, Fernandez N (2018). Structural basis for terminal loop recognition and stimulation of pri-miRNA-18a processing by hnRNP A1. Nat Commun.

