# Supplementary material for: Chronic myelogenous leukemia cells remodel the bone marrow niche via exosome-mediated transfer of miR-320
Source: Theranostics. 2019 Jul 28;9(19):5642–56. doi: 10.7150/thno.34813 (PMC6735391; doi:10.7150/thno.34813)
Supplement: Supplementary file 1 — Supplementary figures and tables. [file thnov09p5642s1.pdf]

**Supplementary information**

# Chronic myelogenous leukemia cells remodel the bone marrow niche via exosome-mediated transfer of miR-320

Xiaotong Gao[1#], Zhuo Wan[1#], Mengying Wei[2,3#], Yan Dong[1], Yingxin Zhao[1], Xutao Chen[4], Zhelong Li[5], Weiwei Qin[1*], Guodong Yang[2,3*], Li Liu[1,*]

[1]Department of Hematology, Tangdu Hospital, Fourth Military Medical University, Xi'an, 710038, People's Republic of China.

[2]State Key Laboratory of Cancer Biology, Fourth Military Medical University, Xi'an, 710032, People's Republic of China.

[3]Department of Biochemistry and Molecular Biology, Fourth Military Medical University, Xi'an, 710032, People's Republic of China.

[4]Department of Implantation, School of Stomatology, Fourth Military Medical University, Xi'an, 710032, People's Republic of China.

[5]Department of Ultrasound Diagnostics, Tangdu Hospital, Fourth Military Medical University, Xi'an, 710038, People's Republic of China.

[#] These authors contributed equally to this work.

*Correspondence and request for materials should be addressed to Li Liu (email: heamatol@fmmu.edu.cn). Correspondence may also be addressed to Guodong Yang (email: yanggd@fmmu.edu.cn) and Weiwei Qin (email: vivianq1126@126.com)

**All the authors declare no potential conflicts of interest.**

## Supplementary Materials and Methods

### Fluorescently labeling of exosomes for in vitro and in vivo tracing

For in vitro analysis of the uptake of leukemic exosomes, exosomes in suspension were labeled with 10 μM DiI (Invitrogen, Carlsbad, CA, USA) in a 37 $^{o}$C water bath for 20 minutes and repelleted by ultracentrifugation at 100,000g for 2 hr. DiI-labeled exosomes were added into culture medium of normal BMMSC, which were plated on round cover slips (Electron Microscopy Sciences, Hatfield, PA, USA) at $2\times10^3/cm^2$, and incubated for another 24 hr. Images of exosome uptake were acquired with a confocal laser scanning microscope.

For in vivo tracking of exosomes, exosomes prepared from cell lines K562, HeLa (human cervical carcinoma), and A549 (human non-small cell lung cancer) were stained with 10 μM DiR (Invitrogen, Carlsbad, CA, USA) similar as the DiI method mentioned above. About 200 μg of DiR-labeled K562, HeLa or A549-derived exosomes were intravenously injected in C57BL/6 mice for 24 hours. The localization of the exosomes in organs was detected by imaging using the IVIS® Lumina II in vivo imaging system (PerkinElmer, Waltham, MA, USA).

Flow cytometry was used to analyze the exosome distribution in different cell populations in the bone marrow. Briefly, 200 μg of DiO-labeled exosomes were intravenously injected in C57BL/6 mice, and bone marrow cells were harvested 24 hours later. DiO positive cells in bone marrow mesenchymal stromal cells (CD45- Ter119- CD31-), endothelial cells (CD45- Ter119- CD31+), granulocyte (Mac1+ Gr1+), B cells (B220+), and T cells (CD3+) were analyzed by flow cytometry.

### Flow cytometric analysis

Single cell suspensions from bone marrow, spleen, and peripheral blood were analyzed by flow cytometry using antibodies listed in the supplemental materials (Table S2). All samples were first stained for 20 min at 4 $^{o}$C with CD16/32 Fc blocking antibody (Biolegend, San Diego, CA, USA) unless indicated otherwise. Stained cells were collected using Beckman FC500 or BD FACSCalibur and analyzed using FlowJo software (FlowJo, Ashland, OR, USA).

### Western blot

Cells or exosomes were homogenized in lysis buffer (20 mM Tris [pH7.5], 150 mM NaCl, 1% Triton X-100 2 mM sodium pyrophosphate，25 mM β-glycerophosphate) supplemented with Protease Inhibitor Cocktail (Roche). Protein extracts were separated in 10% or 12% SDS-PAGE and transferred to nitrocellulose membrane. The nitrocellulose membrane was blocked with 5% nonfat milk for 1 hr and then incubated overnight with primary antibodies at 4 $^{o}$C. The membrane was then incubated 1 hr with peroxidase-conjugated secondary antibodies at room temperature and visualized using the ECL Prime Western Blotting Detection Reagent (GE Healthcare，Buckinghamshire UK). All the details of antibodies are listed in Table S2.

**Plasmid construction and lentivirus production**

For knockdown of HNRNPA1 in K562 cell line, lentivirus expression vector pLKO.1 was used to express shRNAs for human HNRNPA1 (TRCN0000006583, TRCN0000006586, TRCN0000368907) or scrambled shRNA as control. For overexpression of HNRNPA1, human HNRNPA1 coding sequence was amplified from cDNA of K562 cell line and inserted into the lentivirus expression vector pCDH-CMV-MCS-EF1a-PURO with BamHI and EcoRI sites. The same vector expressing eGFP was used as control.

For overexpression and knockdown of miR-320 in K562 cell line, we used the lentivirus expression vector pLKO.1 to express the mature sequence of miR-320a or a sponge sequence that contain 6 repeats of miR-320 binding sites. Detailed sequences used to generate constructs were listed in Table S3.

Lentivirus was produced by transfecting HEK293T cells with lentivirus expression vector along with psPAX2 and pMD2.G packaging plasmids. Supernatants containing lentivirus particles were collected 48 hr after transfection, filtered and stored. For virus infection, virus containing medium were added to cells, and after 6 hr incubation, medium was changed to RPMI 1640 complete medium for another 48 h. The lentivirus infected cells were selected using 3μg/ml puromycin for 2-4 weeks.

**Cell cycle analysis**

For evaluation of K562 cells' cell cycle distribution, cells were fixed with 70% ice-cold ethanol for 1 h, treated with RNase A (100 μg/ml) for 30 min at 37 $^{o}$C, and then stained with 20 μg/ml

propidium iodide for 30 min at 37 °C. Cells were analyzed with BD FACSCalibur flow cytometer.

**EdU incorporation assay**

EdU was added to the culture medium for a final concentration of 20 μM and incubated at 37 °C for 30 min. Cells were fixed and stained with EdU In Vitro Imaging Kit (Sangon, Shanghai, China), according to manufacturer's instruction. The percentage of EdU positive cells was determined using ImageJ software.

**Cell Counting Kit-8 assay**

Cell proliferation was analyzed using the Cell Counting Kit-8 (CCK-8) (Dojindo, Mashiki, Kumamoto Japan). Briefly, K562 cells with different treatments were plated in 96-well plates at 400/well. The cells were normally cultured for 1 to 7 days before 10 μl CCK-8 reagent was added and incubated for another 2 hours. Absorbance was measured at 450 nm using a SpectraMax 96-well plate reader (Molecular Devices, Sunnyvale, CA, USA).

**Micro-computed tomography (microCT) analysis of bone density**

Distal femoral metaphases were scanned by microCT (eXplore Locus SP, GE Healthcare, Milwaukee, WI, USA) under the following specifications: voltage, 80 kV; current, 80 mA; exposure time, 3000 ms; total rotation, 360°; rotation angle increment, 0.4°; resolution rate, 21 μm. Bone morphometric indices were assessed using 3D image reconstructions, included cortical wall thickness (mm), bone volume relative to tissue volume (BV/TV, %), trabecular thickness (mm), trabecular number (1/mm) and trabecular separation (mm).

## Supplementary Figures

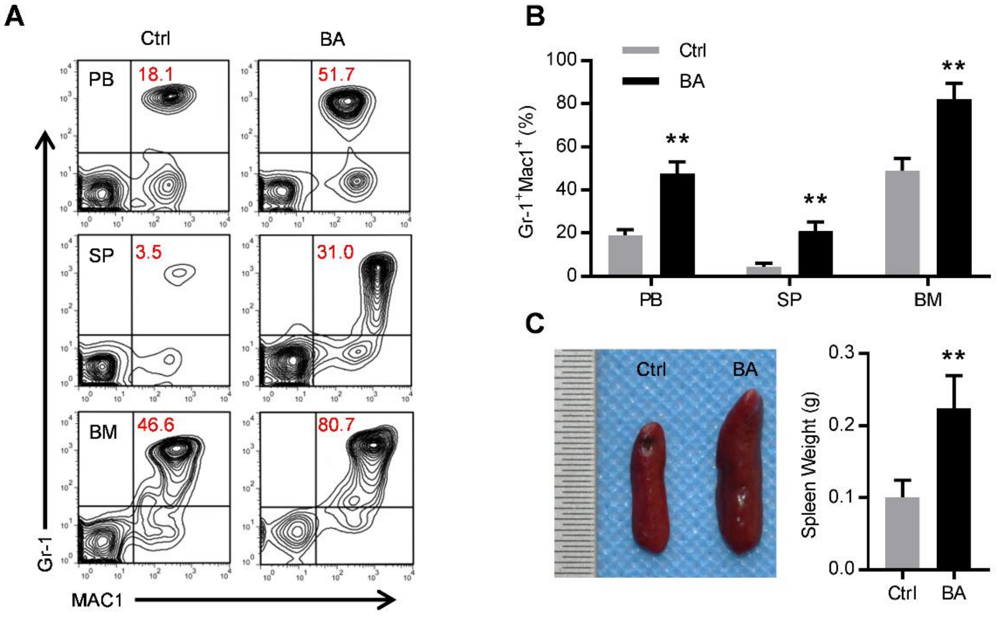


**Figure S1. Phenotyping of control (Ctrl) and CML (BA) mice.** Representative flow cytometry contour plots (A) and average values (B) show the neutrophilic cell percentage detected in peripheral blood (PB), spleen (SP) and bone marrow (BM) of control (Ctrl) and CML (BA) mice. Data are expressed as mean ± SEM (n=6 mice per group). (C) Morphology and weight of spleens from control and CML mice. Data are expressed as mean ± SEM (n=6 mice per group). ** $p$<0.01 by t test. (Corresponding to Figure 1)

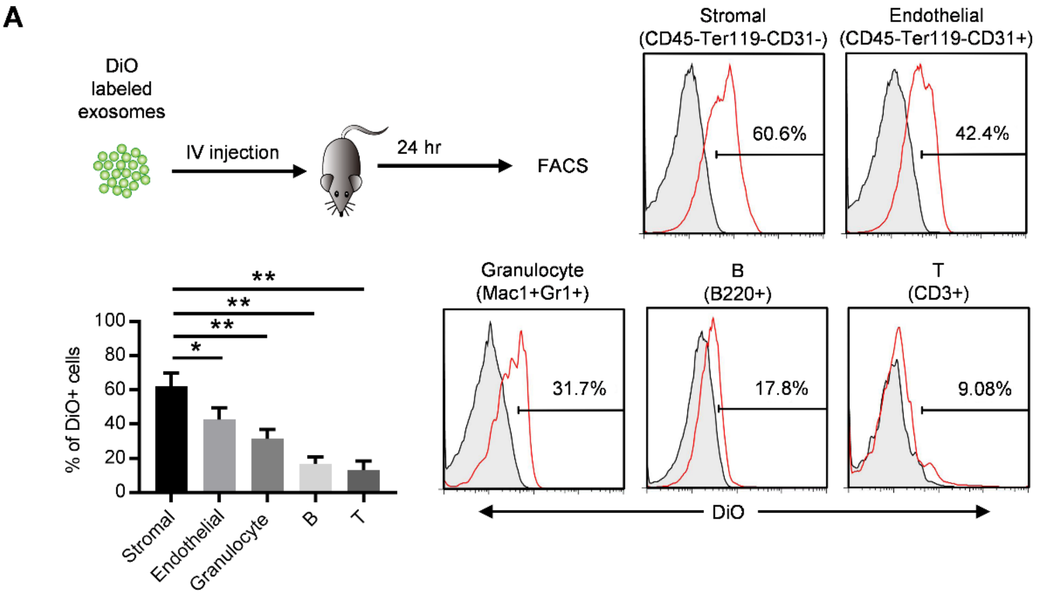


**Figure S2. Distribution of K562 derived exosomes in the cell subpopulation of the bone marrow.** (A) 200 µg DiO-labeled exosomes derived from K562 cells were intravenously injected into C57BL/6 mice. Bone marrow cells were harvested 24 hr later for FACS analysis. Representative images showing the percentage of DiO+ cells within in indicated BM subpopulations, with the grey part indicating the PBS control for DiO- cells. Data are expressed as mean ± SEM (n=3 mice per group). * $p$<0.05, ** $p$<0.01 by one way ANOVA. (Corresponding to Figure 2)

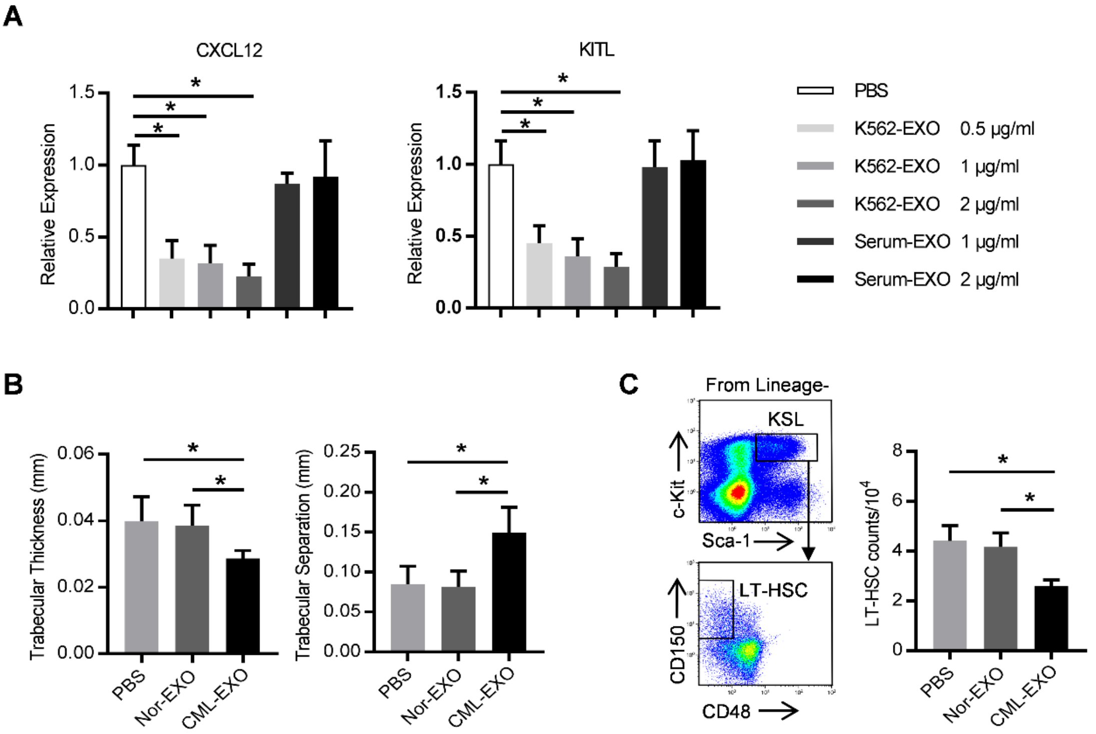


**Figure S3. Effects of exosomes of different origins on the bone marrow niche.** (A) qPCR analysis of genes of interest in human BMMSC treated with indicated concentration of exosomes derived either from leukemic cells or normal serum. PBS-treated BMMSC were used as control. Data are presented as the mean ± SEM of three independent experiments (Corresponding to Figure 3C). (B) Average values of trabecular thickness, and space between trabeculae in trabecular bone region, of mice treated with PBS, Nor-EXO, or CML-EXO, (Nor-EXO: exosomes derived from bone marrow mononuclear cells (BM-MNC) of control normal mice; CML-EXO: exosomes derived from the BM-MNC of BA mice). Mice treated with PBS served as control. Data are expressed as the mean ± SEM (n=6 mice per group), (Corresponding to Figure 3D). (C) Gating strategy and the frequency of long-term hematopoietic stem cell (LT-HSC) in bone marrow of mice treated with PBS, Nor-EXO, or CML-EXO (Corresponding to Figure 3). Data are presented as the mean ± SEM of 8 mice per group. * $p<0.05$, by one way ANOVA.

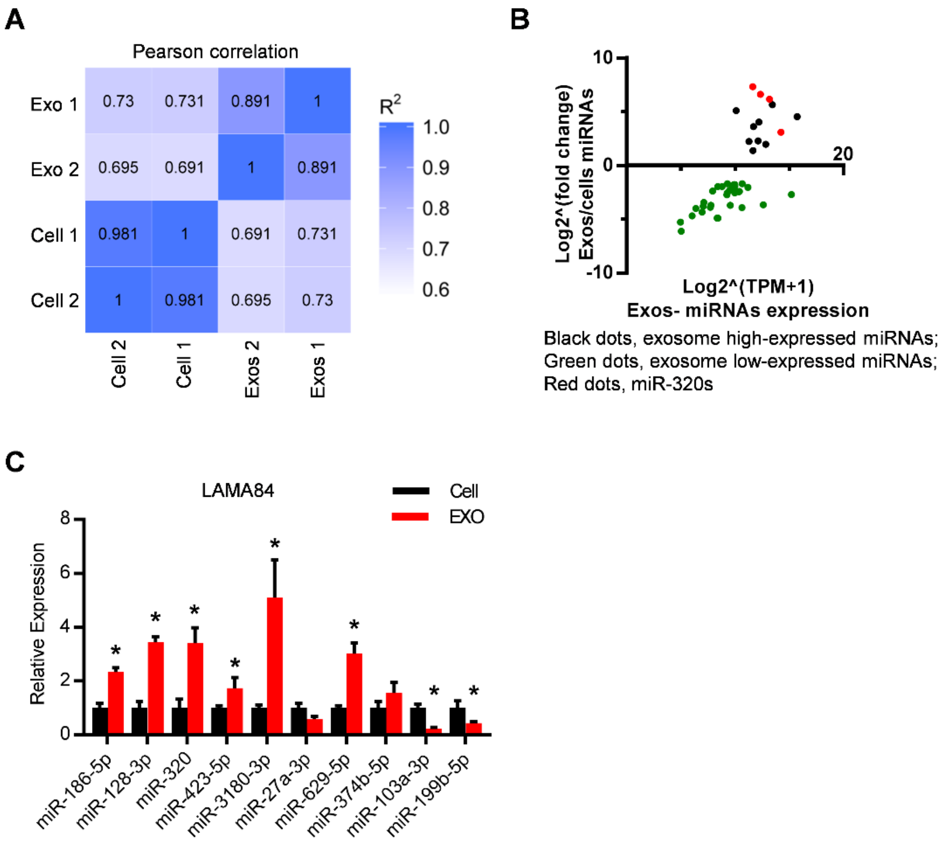


**Figure S4. miRNA profiling in the exosomes and the parental cells.** (A) Pearson correlation analysis of miRNA sequencing data of two replicates of the exosomes and the parental K562 cells. (B) Scatter plot shows the fold change of exosome/cell-enriched miRNAs. Enriched in cells (green), Enriched in exosomes (black and red), miR-320 family (red). (C) Relative expression of indicated miRNAs in both LAMA84 cells and the derived exosomes. Data are presented as the mean ± SEM (n=3 per group). * $p<0.05$ by t test. (Corresponding to Figure 4)

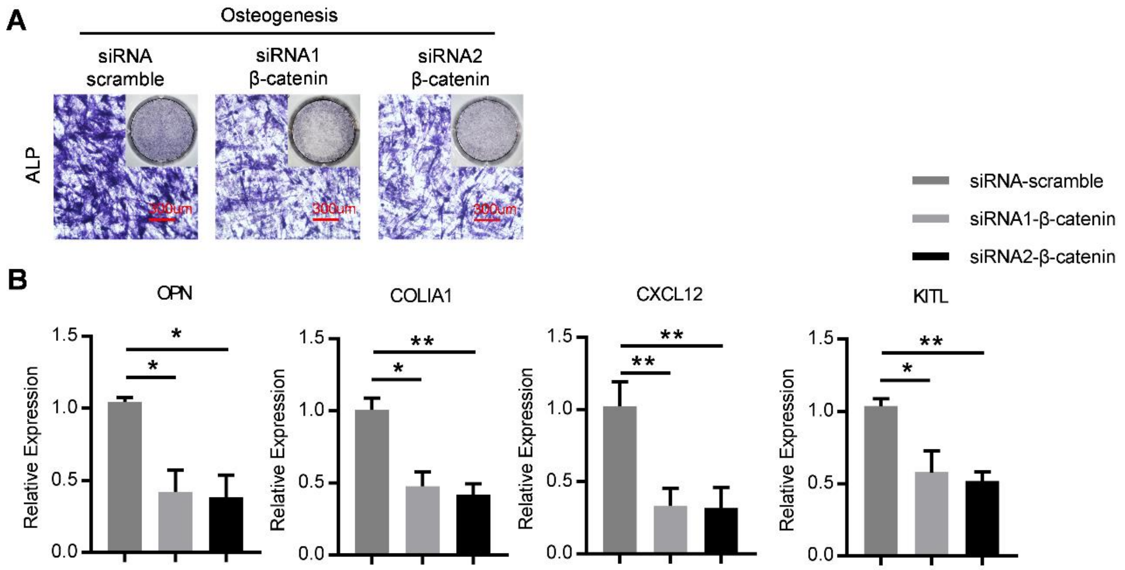


**Figure S5. Effects of β-catenin knockdown on osteogenesis of BMMSC.** (A) ALP staining analysis of the osteogenic differentiation of BMMSC transfected with scramble siRNA or siRNAs against β-catenin and additionally cultured in osteogenic condition for 14 days. Representative images of 3 independent experiments. (B) qPCR analysis of mRNA expression of indicated genes in BMMSC transfected with indicated siRNAs and additionally cultured in osteogenic condition for 3 days. Data are presented as the mean ± SEM of three independent experiments. * $p<0.05$, ** $p<0.01$ by one way ANOVA. (Corresponding to Figure 6)

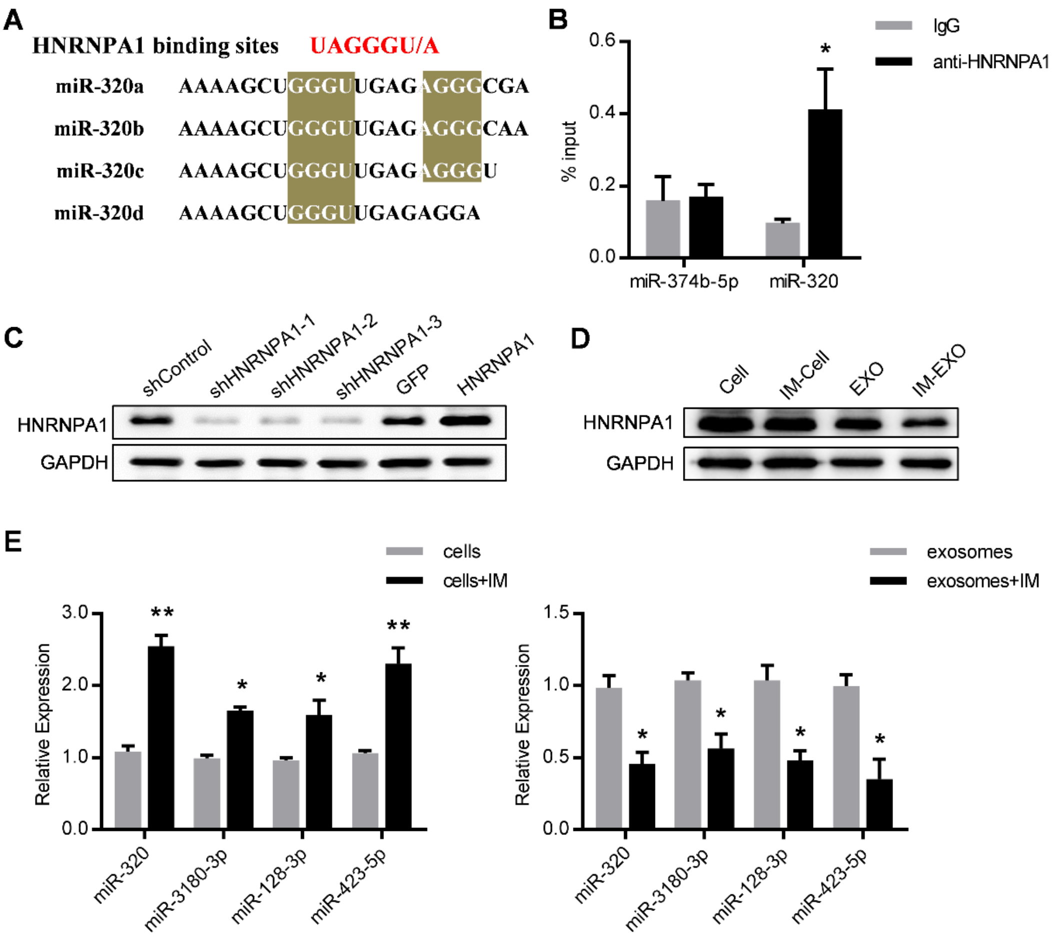


**Figure S6. Effects of Imatinib treatment on HNRNPA1 mediated miR-320 sorting.** (A) Schematic diagram shows the HNRNPA1 binding sites in mature miR-320 (Corresponding to Figure 7). (B) qPCR analysis showing binding of miR-320 by HNRNPA1 in immunoprecipitates from cytoplasmic extracts of bone marrow mononuclear cells from BA mice, with miR-374b-5p serving as control. Data are means ± SEM of three independent experiments (Corresponding to Figure 7D). (C) Western blot analysis of HNRNPA1 knock down or overexpression efficiency (Corresponding to Figure 7E and 7F). (D) Western blot analysis of cellular (Cell) and exosomal (EXO) hnRNPA1 protein level in K562 cells and the derived exosomes treated with or without IM for 48 hours. Images are representative of 3 independent experiments. (E) qPCR analysis of cellular and exosomal miRNAs of K562 cells treated with or without IM for 48 hours. Data are expressed as mean ± SEM of 3 different experiments. * $p$<0.05, ** $p$<0.01 by t test.

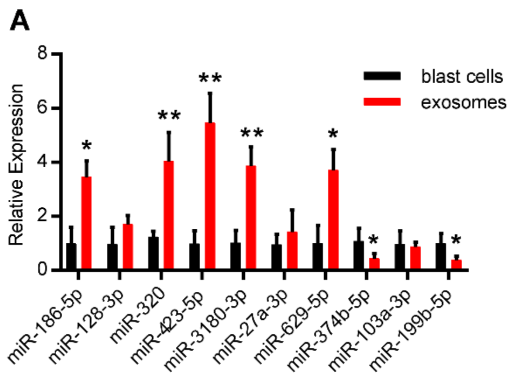


**Figure S7. Abnormal expression of miRNA candidates in clinical CML samples. (A)** qPCR analysis of indicated miRNAs in both cells and exosomes from CML-CP patients. (n=6 patients). (Corresponding to Figure 8). * $p$<0.05, ** $p$<0.01 by t test.

# Supplementary Tables

## Table S1: Primers for qPCR analysis.

| Name | Sequence |
|---|---|
| **GAPDH** | Forward: TGCACCACCAACTGCTTAGC |
| | Reverse: GGCATGGACTGTGGTCATGAG |
| **hnRNPA1** | Forward: GAAAAGCCCTGTCAAAGCAAGAG |
| | Reverse: ACGACCGAAGTTGTCATTCCC |
| **COL1A1** | Forward: GAGGGCCAAGACGAAGACATC |
| | Reverse: CAGATCACGTCATCGCACAAC |
| **OPN** | Forward: CCGAGAAGCTGTGCATCTACAC |
| | Reverse: AGGTTCCACTTGAGCTTGTTCAC |
| **CXCL12** | Forward: ATTCTCAACACTCCAAACTGTGC |
| | Reverse: ACTTTAGCTTCGGGTCAATGC |
| **KITL** | Forward: AGCTTCCAGACACGCTATCAT |
| | Reverse: CGGTACAACGAGCTGTTTCTAC |
| **hsa-miR-186-5p** | CAAAGAATTCTCCTTTTGGGCT |
| **hsa-miR-128-3p** | TCACAGTGAACCGGTCTCTTT |
| **has-miR-320** | AAAAGCTGGGTTGAGAGG |
| **hsa-miR-423-5p** | TGAGGGGCAGAGAGCGAGACTTT |
| **hsa-miR-3180-3p** | TGGGGCGGAGCTTCCGGAGGCC |
| **hsa-miR-27a-3p** | TTCACAGTGGCTAAGTTCCGC |
| **hsa-miR-629-5p** | TGGGTTTACGTTGGGAGAACT |
| **hsa-miR-374b-5p** | ATATAATACAACCTGCTAAGTG |
| **hsa-miR-103a-3p** | AGCAGCATTGTACAGGGCTATGA |
| **hsa-miR-199b-5p** | CCCAGTGTTTAGACTATCTGTTC |
| **mus-miR-320** | AAAAGCTGGGTTGAGAGG |
| **mus-miR-374b-5p** | ATATAATACAACCTGCTAAGTG |
| **U6** | GGATGACACGCAAATTCGTGAAGC |

## Table S2: Antibodies and regents

| Antibodies | Source | Catalogue | Clone |
|---|---|---|---|
| **FACS antibody** | | | |
| Biotin Anti-Mouse CD45 | Biolegend | 103104 | 30-F1 |
| Biotin Anti-Mouse TER-119 | Biolegend | 116204 | TER-119 |
| APC anti-mouse CD31 | Biolegend | 102410 | 390 |
| PE Streptavidin | Biolegend | 405204 | |
| PE Anti-Mouse Ly-6G/Ly-6C (Gr-1) | Biolegend | 108408 | RB6-8C5 |
| APC Anti-Mouse/Human CD11b (Mac1) | Biolegend | 101212 | M1/70 |
| PE anti-mouse/human CD45R/B220 | Biolegend | 103208 | RA3-6B2 |
| PE anti-mouse CD3ε | Biolegend | 100308 | 145-2C11 |
| 7-AAD Viability Staining Solution | Biolegend | 420404 | |

| | | | |
|---|---|---|---|
| FITC anti-mouse Sca-1 | Biolegend | 108106 | D7 |
| APC anti-mouse c-Kit | Biolegend | 105812 | 2B8 |
| PE anti-mouse CD150 | Biolegend | 115903 | TC15-12F12.2 |
| APC/Cy7 anti-mouse CD48 | Biolegend | 103431 | HM48-1 |
| Ultra-LEAF Purified anti-mouse CD16/32 Antibody | Biolegend | 101330 | 93 |
| **Western blot antibody** | | | |
| RUNX2 Rabbit mAb | Cell Signaling Technology | #12556 | D1L7F |
| Anti-beta Catenin Rabbit mAb | abcam | ab32572 | E247 |
| rabbit anti-human CD9 | System Biosciences | EXOAB-CD9A-1 | Polyclonal |
| rabbit anti-human, mouse, rat TSG101 | System Biosciences | EXOAB-TSG101-1 | Polyclonal |
| Rabbit anti human mouse GM130/GOLGA2 | novusbio | NBP2-53420SS | Polyclonal |
| rabbit anti-BCR | Cell Signaling Technology | 3902S | Polyclonal |
| Mouse nnti-hnRNP A1 mAb | abcam | ab5832 | 9H10 |
| rabbit anti-GAPDH mAb | Abcam | ab8245 | 6C5 |
| Goat Anti-Rabbit IgG H&L (HRP) | Abcam | ab6721 | Polyclonal |
| Rabbit Anti-Mouse IgG H&L (HRP) | Abcam | ab6728 | Polyclonal |
| **Regents** | | | |
| DiO | Invitrogen | D275 | |
| DiI | Invitrogen | D282 | |
| UltraGRO-Adavanced | HELIOS | Hpcfdcrl50 | |
| Protector Rnase Inhibitor | Roche | 489703001 | |
| Protease Inhibitor Cocktail Tablets | Roche | 4693159001 | |
| Protein A Sepharose | Abcam | ab193256 | |
| Streptavidin Sepharose Beads | CST | 3419S | |
| CD34 MicroBeads Kit human | Miltenyi Biotec | 130-046-702 | |
| ECL prime western blotting detection reagent | GE healthcare | RPN2232 | Buckinghamshire |
| Mouse Lineage Cell Depletion Kit | Miltenyi Biotec | 130-090-858 | CD5,CD45R(B220) CD11b,Anti-Gr-1(Ly-6G/C),7-4,Ter-119 |

## Table S3: Oligos/Primers used for shRNA cloning into pLKO.1 vector, HNRNPA1 overexpression in pCDH-CMV-MCS-EF1a-PURO, or siRNA target sequence of β-catenin knockdown.

| Name | Sequence |
|---|---|
| **TRCN0000006583** | Forward:CCGGGCCACAACTGTGAAGTTAGAACTCGAGTTCTAACTTCACAGTTGTGGCTTTTTG |
| | Reverse:AATTCAAAAAGCCACAACTGTGAAGTTAGAACTCGAGTTCTAACTTCACAGTTGTGGC |
| **TRCN0000006586** | Forward: CCGGAGATATTTGTTGGTGGCATTACTCGAGTAATGCCACCAACAAATATCTTTTTTG |
| | Reverse: AATTCAAAAAAGATATTTGTTGGTGGCATTACTCGAGTAATGCCACCAACAAATATCT |
| **TRCN0000368907** | Forward: CCGGGCTATAATGGATTTGGTAATGCTCGAGCATTACCAAATCCATTATAGCTTTTTG |
| | Reverse: AATTCAAAAAGCTATAATGGATTTGGTAATGCTCGAGCATTACCAAATCCATTATAGC |
| **miR-320a mimic** | Forward:CGGAAAAGCTGGGTTGAGAGGGCGACTCGAGTCGCCCTCTCAACCCAGCTTTTGTTTTTG |

| | |
|---|---|
| | Reverse:AATTCAAAAACAAAAGCTGGGTTGAGAGGGCGACTCGAGTCGCCCTCTCAACCCAGCTTTT |
| **miR-320a sponge** | ACCGGTGCTCGCTTCGGCAGCACATATACTAGTCGACGCGGCCGCCTCGAGCCGGTCGCCCTCTCCTAC AGCTTTTCCGGTCGCCCTCTCCTACAGCTTTTCCGGTCGCCCTCTCCTACAGCTTTTCCGGTCGCCCTCT CCTACAGCTTTTCCGGTCGCCCTCTCCTACAGCTTTTCCGGTCGCCCTCTCCTACAGCTTTTCCGGTACC GGATCGCGGGCCCGTTTAAACCCGCTCTAGAGCGGACTTCGGTCCGCTTTTT GAATTC |
| **HNRNPA1 overexpression** | HNRNPA1-BamHI-Forward: CGGGATCCATGTCTAAGTCAGAGTCTCC<br>HNRNPA1-EcoRI-Reverse: GCGAATTCTTAAAATCTTCTGCCACTGC |
| **siRNA1-β-catenin** | ATCTGTCTGCTCTAGTAATAA |
| **siRNA2-β-catenin** | GCTTGGAATGAGACTGCTGAT |

**Table S4: Examples of high-throughput mass spectrometry analysis of microRNA precipitated proteins**

| | Biotin-miR-320a | Biotin-miR-374b-5p | Beads |
|---|---|---|---|
| hnRNP A1 | 8 | 3 | 0 |
| hnRNP A2/B1 | 6 | 5 | 0 |
| hnRNP M | 4 | 6 | 0 |
| hnRNP L | 3 | 3 | 0 |
| IGF2BP2 | 1 | 1 | 0 |
| HSP90AB1 | 2 | 3 | 0 |
| RPL26 | 2 | 1 | 0 |

**Notes**: hnRNP heterogeneous nuclear ribonucleoprotein
IGF2BP2 Insulin-like growth factor 2 mRNA-binding protein 2
HSP90AB1 Heat shock protein HSP 90-beta
RPL26 60S ribosomal

**Table S5: Motif analyses of miRNAs enriched in cells and exosomes**

| **miRNAs enriched in cells** | **log2 Fold Change** | **hnRNP A1 binding sites** | **Biological function** |
|---|---|---|---|
| hsa-miR-374b-5p | -6.0404 | | TS |
| hsa-miR-374c-3p | -6.039 | | - |
| hsa-miR-106b-5p | -5.3423 | | onco |
| hsa-miR-103b | -4.8181 | | - |
| hsa-miR-103a-3p | -4.8038 | AGGG | onco |
| hsa-miR-199b-5p | -4.6507 | | onco |
| hsa-miR-30b-5p | -4.3187 | | onco |
| hsa-miR-18a-5p | -4.0856 | | onco |
| hsa-miR-17-5p | -3.9305 | | onco |
| hsa-miR-26b-5p | -3.9053 | UAGG | TS |
| hsa-miR-142-3p | -3.7537 | | onco |
| hsa-miR-340-5p | -3.7426 | | TS |
| hsa-miR-101-3p | -3.7211 | | TS |
| hsa-miR-20a-5p | -3.644 | | onco |
| hsa-miR-146b-3p | -3.453 | | onco |

| | | | |
|---|---|---|---|
| hsa-miR-452-5p | -2.7954 | | onco |
| hsa-miR-21-5p | -2.7443 | | onco |
| hsa-miR-140-3p | -2.6517 | AGGG | TS |
| hsa-miR-148b-3p | -2.5934 | | onco |
| hsa-miR-3529-3p | -2.4934 | | - |
| hsa-miR-7-5p | -2.4804 | | TS |
| hsa-miR-126-5p | -2.4396 | | TS |
| hsa-miR-185-5p | -2.3717 | | onco |
| hsa-miR-93-5p | -2.3692 | | onco |
| hsa-miR-182-5p | -2.1047 | | onco |
| hsa-miR-16-5p | -2.0501 | | TS |
| hsa-miR-30e-3p | -2.0338 | | - |
| hsa-let-7f-5p | -2.024 | | TS |
| hsa-miR-30c-5p | -1.9898 | | TS |
| hsa-miR-30e-5p | -1.8184 | | onco |
| hsa-miR-19b-3p | -1.8114 | | onco |
| hsa-miR-27b-3p | -1.7831 | | TS |
| hsa-miR-425-5p | -1.7288 | | onco |
| **miRNAs enriched in exosomes** | | | |
| hsa-miR-320d | 7.1207 | AGGG | TS |
| hsa-miR-320c | 6.4668 | AGGG | TS |
| hsa-miR-320b | 6.0608 | AGGG | TS |
| hsa-miR-1246 | 5.5058 | | onco |
| hsa-miR-10b-5p | 4.7278 | | onco |
| hsa-miR-3184-3p | 4.3806 | AGGG | - |
| hsa-miR-423-5p | 4.3806 | AGGG | TS/onco |
| hsa-miR-22-3p | 3.8776 | | TS |
| hsa-miR-3180-3p | 3.5397 | | - |
| hsa-miR-320a | 3.0343 | AGGG | TS |
| hsa-miR-629-5p | 2.2368 | GGGA | onco |
| hsa-miR-27a-3p | 2.1821 | | onco |
| hsa-miR-128-3p | 1.9085 | | TS |
| hsa-miR-186-5p | 1.3067 | | TS |

onco: oncogene; TS: tumor suppression.

**Table S6: Biotinylated RNA oligonucleotides used in pull down assay**

| Name | Oligonucleotides sequence |
|---|---|
| **Biotin-miR-320a** | Biotin-CCCUCUUAAAAAGCUGGGUUGAGAGGGCGA |
| **Biotin-miR-374b-5p** | Biotin-CCCUCUUAAUAUAAUACAACCUGCUAAGUG |

**Note:** Underlined nucleotides denote the 8-nt linker.
